# Revealing the structural evolution of CuAg composites during electrochemical carbon monoxide reduction

**DOI:** 10.1038/s41467-024-49158-4

**Published:** 2024-06-01

**Authors:** Di Wang, Hyun Dong Jung, Shikai Liu, Jiayi Chen, Haozhou Yang, Qian He, Shibo Xi, Seoin Back, Lei Wang

**Affiliations:** 1 Department of Chemical and Biomolecular Engineering, College of Design and Engineering, National University of Singapore, Singapore, Singapore; 2 Department of Chemical and Biomolecular Engineering, Institute of Emergent Materials, Sogang University, Seoul, Republic of Korea; 3 Department of Materials Science and Engineering, College of Design and Engineering, National University of Singapore, Singapore, Singapore; 4 Institute of Sustainability for Chemicals, Energy and Environment (ISCE2), Agency for Science, Technology and Research (A*STAR), Singapore, Singapore; 5 Centre for Hydrogen Innovations, National University of Singapore, Singapore, Singapore

**Keywords:** Electrocatalysis, Electrocatalysis

## Abstract

Comprehending the catalyst structural evolution during the electrocatalytic process is crucial for establishing robust structure/performance correlations for future catalysts design. Herein, we interrogate the structural evolution of a promising Cu-Ag oxide catalyst precursor during electrochemical carbon monoxide reduction. By using extensive in situ and ex situ characterization techniques, we reveal that the homogenous oxide precursors undergo a transformation to a bimetallic composite consisting of small Ag nanoparticles enveloped by thin layers of amorphous Cu. We believe that the amorphous Cu layer with undercoordinated nature is responsible for the enhanced catalytic performance of the current catalyst composite. By tuning the Cu/Ag ratio in the oxide precursor, we find that increasing the Ag concentration greatly promotes liquid products formation while suppressing the byproduct hydrogen. CO_2_/CO co-feeding electrolysis and isotopic labelling experiments suggest that high CO concentrations in the feed favor the formation of multi-carbon products. Overall, we anticipate the insights obtained for Cu-Ag bimetallic systems for CO electroreduction in this study may guide future catalyst design with improved performance.

## Abstract (web summary)

Revealing catalyst structural evolution during catalysis is critical. Here, authors reveal that a Cu-Ag oxide precursor undergoes a transformation during CO electroreduction to a composite consisting of Ag nanoparticles enveloped by thin layers of amorphous Cu, which is likely the real active phase.

## Introduction

Electrochemical CO_2_ reduction (CO_2_R) coupled with renewable electricity has been recognized as a promising route for mitigating the pressing carbon emission and enabling the sustainable production of value-added chemicals^1–8^. Mechanistically, CO is the key reaction intermediate for the conversion of CO_2_ to valuable multi-carbon (C_2+_) products during CO_2_R^9^. Thus, great efforts have been made to the investigation of electrochemical CO reduction (COR) aiming to uncover the levers (i.e., catalyst structure, composition, microenvironment) that control the selectivity and activity towards C_2+_ products^10–18^. On the other hand, tremendous success has been achieved for the development of efficient catalysts for CO_2_R to CO^19–26^, offering opportunities for converting CO_2_ to C_2+_ products via a cascade approach (CO_2_ → CO → C_2+_). Additionally, direct COR can effectively mitigate the severe issues associated with carbonate formation during CO_2_R and lead to improved carbon utilization efficiency and system stability of the process. Therefore, there is a need to continue develop COR with the goal of achieving maximized activity and selectivity toward the desired products.

To date, Cu-based materials are the only known catalysts that exhibit appreciable activity and Faradaic Efficiencies (FE) in producing C_2+_ products during COR^27,28^. Despite their potential, the unsatisfactory performance of Cu catalysts under practical relevant conditions has hindered the practical implementation of this technology. A handful of strategies have been explored to modify the physicochemical properties of Cu catalysts, such as controlling the particle size, morphology, facet, grain boundaries and compositions, intending to develop catalysts with improved activity and selectivity for COR. Increasingly, bimetallic materials formed by introducing a second metal to Cu are being explored as a promising approach to tune the activity and selectivity of Cu catalysts for COR. For instance, bimetallic catalysts such as CuAu^29^, CuPd^30,31^ and CuAg^32–35^ have been investigated for their potential for efficient COR. Particularly, CuAg-based materials have received significant attentions owning to their promising performance towards COR and CO_2_R^36–42^. In these researches, phase separation and alloy represent two forms of CuAg materials. In phase-separated CuAg materials, there is a clear interface between Cu/Ag, which is considered as active sites of the reaction^32,42^. In CuAg alloy, a small amount of Ag is doped into the Cu lattice, and the Cu atoms around Ag are claimed to promote the production of C_2+_^37^.

**Fig. 1:**
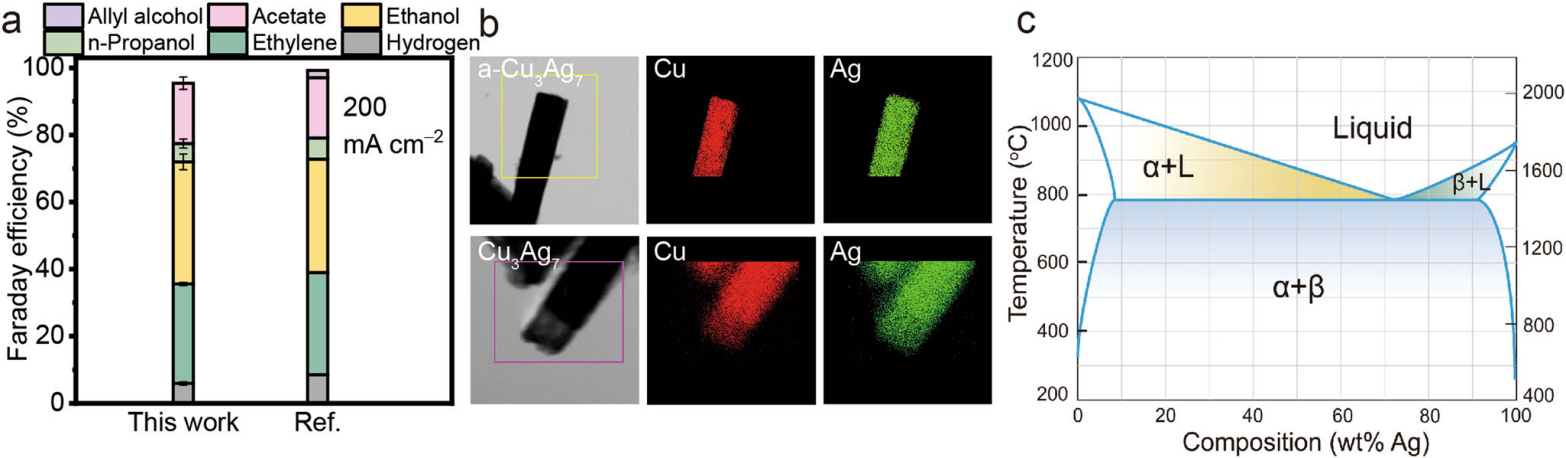
COR performance on CuAg and structural information of Cu and Ag. **a** Faradaic efficiency (FE) of the CuAg samples in this work and reference work^34^. **b** XEDS elemental mapping of as-prepared Cu/Ag precursors and post-reacted CuAg catalysts. **c** Phase diagram of CuAg. The error bars represent standard deviations from at least three independent measurements. Relevant source data are provided as a Source Data file.

During the preparation of our manuscript, a few Ag-modified oxide-derived Cu catalysts were recently reported elsewhere showing improved FEs toward C_2+_ liquid products in COR^32–34^. We also prepared Cu/Ag mixed-oxide precursors and employed them for COR, as the Cu and Ag atoms are initially well mixed within the precursors. The catalysts in our work are labeled based on the atomic ratios of Cu and Ag employed during preparation. For instance, Cu_3_Ag_7_ represents an atomic ratio of 3:7 between Cu and Ag. Besides, a-Cu_3_Ag_7_ denotes the as-prepared Cu_3_Ag_7_ precursor. Inductively Coupled Plasma Optical Emission spectroscopy (ICP-OES) indicate consistent Cu to Ag ratios in post-COR samples compared to the corresponding as-prepared samples (Supplementary Table 1). As shown in Fig. 1a, the CuAg catalysts used in our study and the previous work exhibit nearly identical selectivity at similar COR current densities, i.e., high FEs of >90% towards C_2+_ products were achieved with 60% of which being liquid products including ethanol, acetate and propanol^34^. Additionally, the catalysts were found to both have uniform distributions of Cu and Ag within oxide precursors, as shown in the X-ray Energy Dispersive Spectroscopy (XEDS) elemental map (Fig. 1b). Notably, no obvious segregations of Ag or Cu were observed for both catalysts even after extended period of COR reactions (Supplementary Figs. 4 and 5). At first glance, this may lead to the conclusion of that the active phase of this bimetallic catalyst is the solid solution of Cu and Ag. However, it is contradictory to the immiscibility nature of Cu and Ag (Fig. 1c), i.e., the solubility of Cu in Ag or vice versa are largely illegible at temperatures below 300 °C^43^. Therefore, we hypothesis that the Cu and Ag co-exist in other forms post COR, such as core-shell type of structures, induced by significant structural evolutions of the Cu/Ag oxide precursors during COR. Additionally, CuAg catalysts with higher Ag contents exhibited increased selectivity towards C_2+_ products, suggesting that the COR selectivity is closely related to the structure formed by CuAg with different Cu/Ag ratio. Overall, the above surprising observations have motivated us to carefully explore the detailed structural evolution of the Cu/Ag oxide precursors during COR, and determine the true active phase that are responsible for the high selectivity and activity towards C_2+_ products.

In this work, comprehensive physical characterizations, including scanning transmission electron microscopy (STEM), *operando* X-ray absorption fine structure spectroscopy (XAFS), X-ray diffraction analysis (XRD) and X-ray photoelectron spectroscopy (XPS) were employed to track the dynamic structural evolution of Cu/Ag oxides during COR. Our findings suggest that Cu/Ag oxide precursors transformed to phase-separated Cu and Ag metallic states during COR under practical relevant conditions. Taking the Cu/Ag oxide with optimized composition (Cu_3_Ag_7_) as an example, both the Cu and Ag oxides were reduced into metallic forms during COR. The bimetallic material underwent structural transformation and formed small Ag particles wrapped with thin and amorphous Cu layers. Our computational investigations based on Density Functional Theory (DFT) indicate that the amorphous Cu on top of Ag lattice exhibits stronger bindings towards CO^*^ and CH_3_CHO^*^, which lead to the favorable formation of C_2+_ products, i.e., ethanol. Furthermore, through CO_2_/CO co-feeding electrolysis together with isotopic labeling, we identified that most of C_2+_ products were derived from CO rather than CO_2_, consistent with recent findings reported elsewhere^44^. Specifically, the introduction of CO gas feeds resulted in an increased formation of C_2+_ products, indicating that under our experimental conditions, COR exhibits higher activity for C_2+_ products formation of compared to CO_2_R. This highlights the advantages of conducting COR. Notably, in CuAg samples, this phenomenon is further enhanced with the addition of Ag. Overall, our study reveals that the Ag-induced amorphous Cu is the origin of the improved selectivity and activity towards C_2+_ products, particularly liquid products. These insights will contribute to our understanding of the mechanism of COR and the design strategies for catalysts.

## Results

### Physical characterizations of Cu/Ag oxides before and after COR

**Fig. 2:**
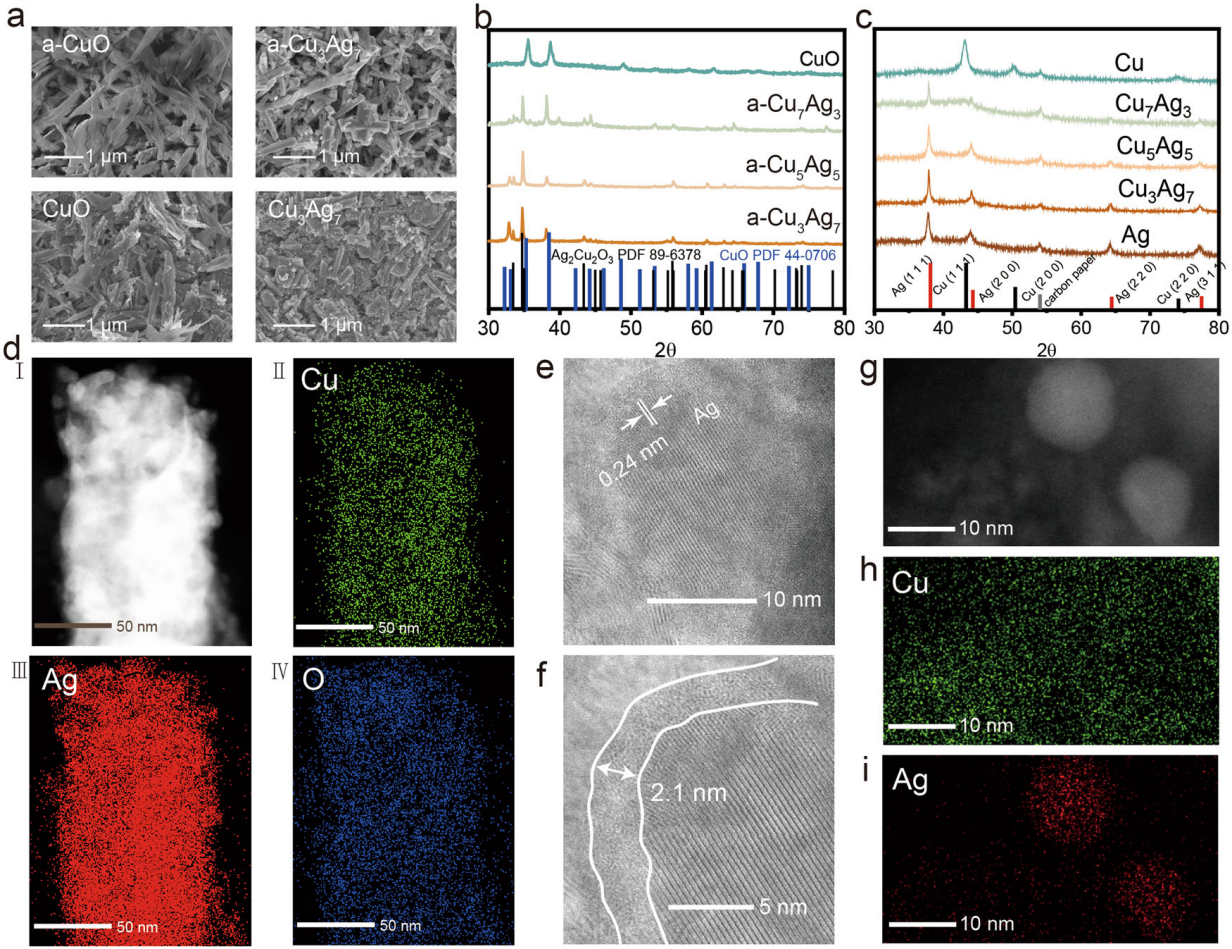
Structural characterization of CuO and Cu_3_Ag_7_ catalysts before and after COR at 200 mA cm^2^. **a** SEM images of as-prepared CuO (a-CuO) and Cu_3_Ag_7_ (a-Cu_3_Ag_7_) precursors and post-COR CuO and Cu_3_Ag_7_ samples. **b** XRD patterns of as-prepared CuO and CuAg precursors. **c** XRD patterns of post-reacted CuO and CuAg catalysts. **d** STEM XEDS elemental mapping of post-reacted Cu_3_Ag_7_ catalyst. **e**, **f** HRTEM images of rod-shaped particles edge in post-reacted Cu_3_Ag_7_. **g** STEM image of nanoparticles on the surface of post-reacted Cu_3_Ag_7_. **h**, **i** STEM XEDS elemental mapping of nanoparticles on the surface of post-reacted Cu_3_Ag_7_. Relevant source data are provided as a Source Data file.

The typical Cu/Ag oxide precursors and the pristine CuO control samples were prepared according to a modified co-precipitation approach^34^. As depicted by the Scanning Electron Microscope (SEM) images (Fig. 2a and Supplementary Figs. 1 and 2), the as-prepared CuO exhibit flaky morphology. Upon the introduction of Ag, rod-shaped particles with diameters ranging from 500 nm to 2 µm become dominant in the CuAg oxides. After conducting the COR measurement, no significant morphological changes were observed in any of these samples. However, small particles with diameters of approximately 10 nm were discovered to have accumulated on the surface of these samples. This phenomenon could be attributed to the dissolution of Cu and/or Ag in the electrolyte under open circuit voltage (OCV), followed by their redeposition back to the catalyst surface^45,46^. XRD was employed to investigate the crystallographic structures of catalysts before and post COR. Figure 2b demonstrates the composition of the as-prepared precursors, which comprised CuO and Ag_2_Cu_2_O_3_. Notably, Ag_2_Cu_2_O_3_ is a singular Cu/Ag compound, characterized by a uniform distribution of Cu and Ag, as shown in Fig.1b, however, with distinct oxidation states^47–49^. This is consistent with our observation in Fig. 1b. However, out of our expectation, the XRD spectra of the post-COR Cu/Ag samples only show the patterns of metallic Ag (Fig. 2c), while no Cu-related peaks were detected. Hence, drastic structural evolution must have taken place in these CuAg oxide samples during COR. To interpret this phenomenon, we have contemplated three potential scenarios. Firstly, we speculate that Cu may be largely dissolved in the KOH electrolyte during the COR electrolysis. However, XEDS analysis in Fig. 2d reveals that Cu could be easily detected in the post-COR sample, indicating that majority of the Cu content retained during COR. This has been corroborated by the subsequent XPS data (vide infra). Second, Cu existed in the post-COR sample in the form of extremely small clusters, which led to a significant reduction of its X-ray diffraction peak intensity. However, the persistence of small Cu clusters without aggregation during COR appears unlikely due to the low cohesive energy of Cu. Besides, there were no evidences found to substantiate this assumption. Lastly, we hypothesize that the CuO within the Cu/Ag precursor was reduced to metallic Cu and underwent a structural transformation into an amorphous state under the conditions of COR^50–52^. The XRD of Cu_3_Ag_7_ samples after a short period of COR under 200 mA cm^−2^ was also conducted (Supplementary Fig. 12b). Within 10 min of COR, all Cu-related peaks disappeared, indicating the rapid formation of amorphous Cu.

To verify this hypothesis, we conducted post-COR STEM to carefully examine the morphological structure and composition of the CuAg samples. Taking Cu_3_Ag_7_ as an example, its mesostructured and elemental composition retained after COR (Fig. 2d). The utilization of high-resolution STEM with atomic sensitivity enabled us to observe the sample’s composition with exceptional clarity. As shown in Fig. 2e, small Ag nanoparticles exist in the post-COR sample, exhibiting an interplanar spacing of approximately 0.24 nm, corresponding to the Ag (1 1 1) crystal plane^53^. Remarkably, the Ag particles were found to be enveloped in an amorphous layer with an average thickness of ~2 nm (Fig. 2f). The composition of this layer was further confirmed to be Cu by the corresponding XEDS elemental mapping (Supplementary Fig. 6), which agrees well with the above XRD analysis. Drawing upon previous research, this amorphous Cu layer can be attributed to in situ self-reconstruction induced electroactive amorphous species (ISIA)^54–60^. Such layers can emerge during electrocatalysis as a consequence of factors like potential driving, lattice distortion, and defect induction, etc. The formation of ISIA often correlated with changes in electrocatalytic activity that deserves further study^61,62^. For instance, crystalline In_2_O_3_ was reconstructed to crystalline In and amorphous In_2_O_3−x_ during CO_2_R, showing enhanced FE (~90%) towards formate^56^. Moreover, chlorine doping induced the formation of amorphous LiCoO_1.8_Cl_0.2_, showcasing enhanced activity and stability in oxygen evolution reaction (OER)^57^. Previously, it has been reported that Cu atoms show higher mobility in CuAg materials under CO_2_R conditions, potentially leading to their migration toward the electrode surface^42,63^. Since atomic transfer mechanism is considered as a major contributor to the formation of ISIA, we hypothesize that Cu atoms will migrate to CuAg surface under COR condition. For instance, the reduction of oxidized Cu, and the redeposition of the dissolved Cu in alkaline electrolyte, i.e., KOH used in this work. Besides, since Cu and Ag are difficult to form a uniform alloy under normal conditions^64^, the high Ag content in the CuAg samples likely induce lattice mismatches and results in a spatial strain effect between Cu and Ag^65^, which disrupt the formation of crystalline Cu, and causing amorphization of surface Cu. Taken together, we believe the combined effects of cathodic potential during COR, redeposition process of Cu and lattice mismatch induced by Ag lead to the formation of amorphous Cu layers around the Ag particles.

In addition, the STEM images (Fig. 2f) clearly show the interface between Cu and Ag, indicating that Cu and Ag are phase segregated. Such arrangement of Cu and Ag is consistent with the apparent “uniform distributions” of Cu and Ag elements as suggested by the XEDS elemental mappings in this study (Fig. 2g–i) and in previous work^34,66^. In all, we believe that the homogenous Cu/Ag oxide precursors undergo a transformation to a bimetallic composite consisting of small Ag nanoparticles enveloped by thin layers of amorphous Cu under the COR conditions.

### Tracking the dynamic structural evolution of CuAg during COR

**Fig. 3:**
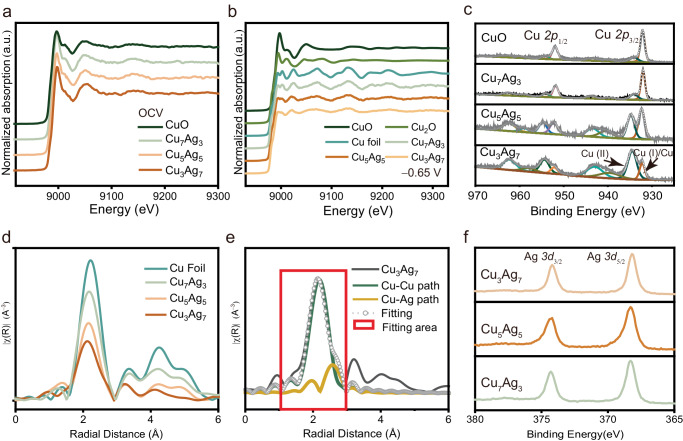
XPS and *Operando* XAFS spectra of CuO and CuAg samples. **a** Cu K-edge XANES and EXAFS spectra of the CuO and CuAg samples at OCV. **b** Cu K-edge XANES and EXAFS spectra of CuAg samples at −0.65 V vs RHE ( ~ 150 mA cm^−2^), Cu_2_O, CuO and standard Cu foil. **d** FT of EXAFS spectra of CuAg samples at −0.65 V vs RHE and standard Cu foil. **e** EXAFS fitting curves at the Cu–Cu path and Cu-Ag path for Cu_3_Ag_7_ at −0.65 V vs RHE. **c**, **f** XPS spectra of CuO and CuAg samples after COR with 200 mA cm^−2^ of (**c**) Cu *2p* and (**f**) Ag *3d*. Relevant source data are provided as a Source Data file.

*Operando* measurements based on XAFS were employed to further explore the structural evolution of the CuAg catalyst during COR. To ensure the relevance of the *operando* XAFS experiments, identical flow cell (Supplementary Fig. 8) and COR conditions (i.e., CO feeding rate, electrolyte, electrode potential, etc.) were employed for the XAFS experiments. In a typical experiment, both the X-ray absorption near-edge structure (XANES) and extended X-ray absorption fine structure (EXAFS) of Cu K-edge were measured for the pristine CuO and various CuAg samples at Open Circuit Voltage (OCV) and −0.65 V vs. RHE, respectively. As shown in Fig. 3a, the Cu-XANES spectra suggest that the chemical states of Cu in these samples are in +2 oxidation state (CuO) under the OCV condition^67–69^, consistent with the above XRD and XPS results. When a negative potential was applied to drive COR, the Cu K-edge absorption edge positions for all samples decreased from 8976 to 8974 eV (Fig. 3b), indicating the valence state of Cu decreased during COR. Since the Cu absorption edge positions of all CuAg samples are nearly identical to that of the standard Cu foil, we believe that the valence state Cu was reduced to its metallic state for all CuAg samples under COR conditions. Rather than valence state, EXAFS fingerprints provide coordination information of the CuAg samples during COR^70,71^. As shown in Fig. 3b, the two peaks located at around 9000 eV in the CuAg samples exhibited notable differences compared to the spectrum of standard Cu foil. Specifically, as the Ag concentration increased, the peak at 9002 eV became flatter, and the peak at 9025 eV shifted towards lower binding energies. To closely examine the atomic structure of CuAg, the Fourier transform (FT) of EXAFS under reaction conditions is analyzed. As shown in Fig. 3d, in the R space, the intensity of the peak at ~2.4 Å decreases with the increasing Ag content, indicating a reduction in the coordination number of Cu within the CuAg samples^72^. In addition, we conducted curve fitting for the FT-EXAFS spectra of Cu_3_Ag_7_ to extract detailed information regarding the neighboring atomic species, coordination number and distance (Fig. 3e). The fitting results revealed that in Cu_3_Ag_7_, the average coordination number of Cu is ~7.6, comprising 6.1 Cu–Cu bonds and 1.5 CuAg bonds (Supplementary Table 6). The total coordination number, which is lower than the typical 12 found in fcc metals like Cu foil for comparative analysis, may stem from the amorphous characteristic of the Cu layer and the immiscibility nature of Cu and Ag. Furthermore, the FT-EXAFS results of CuAg samples in Fig. 3d also demonstrate that with the addition of Ag, the structure of Cu in CuAg undergoes significant evolution. Specifically, the intensity of the peak located at around 4.3 Å decreases significantly with the increase in Ag content within the CuAg samples, indicating a reduction in long-range order of Cu atom arrangement^73,74^. Combining observations from both STEM and XRD analyses, we believe that this reduction in long-range order of Cu can be attributed to both the incorporation of Ag foreign atoms, as well as the formation of amorphous Cu layer. This is supported by the substantially decreased coordination number of Cu in Cu_3_Ag_7_ (Supplementary Table 6). Notably, amorphous Cu was observed solely in CuAg samples with high Ag contents (Fig. 2e, f). Besides, there are no XRD patterns related to crystalline Cu observed in the post-COR CuAg samples (Fig. 2c), while pristine CuO sample did not exhibit such behavior. Hence, we hypothesize that the large Ag content exist in CuAg samples likely plays a role in the formation of amorphous Cu during COR.

XPS was also employed to determine the surface composition of the samples before and after COR. As anticipated, both Ag and Cu in the precursors predominantly exist in their respective oxidation states prior to COR (Supplementary Fig. 13). The labeled Cu *2p*_3/2_ peak in Fig. 3c is corresponding to Cu (I)/Cu for post-reacted CuO and CuAg samples (Supplementary Fig. 14). Thus, after COR, the bulk CuO catalyst predominately exhibits the form of metallic Cu and Cu (I) (Fig. 3c). The presence of Cu (I) and Cu (II) species can be attributed to the native oxide layers formed during the XPS sample preparation. Notably, upon the introduction of Ag, the post-COR CuAg samples show an increased portion of the Cu (II) species, and this increase scales with Ag content within the bimetallic materials (Fig. 3c). Considering that each sample was subjected to identical post-reaction treatments for XPS, this observation is likely attributable to the presence of the thin layer of amorphous Cu within the CuAg samples. This thin layer renders the Cu more prone to oxidation upon exposure to air. In the contrary, as shown in Fig. 3f, Ag within all CuAg samples was reduced to the metallic state. Furthermore, there was no discernible peak position shift for Ag across different samples, indicating a consistent Ag state across the various CuAg samples. Nevertheless, we believe the presence of Ag substantially influences the structural evolution of Cu in CuAg materials, i.e., Cu transition to an amorphous state during COR, especially with higher Ag content.

The dynamic structural evolution of the catalyst during COR/CO_2_R will result changes in product selectivity^75–77^, thus, the structural evolutions of various CuAg catalyst precursors with different Cu/Ag ratios were investigated through XRD, STEM, and *operando* XAFS (Supplementary Fig. 12). Specifically, COR tests with reaction time ranging from 10 min to 10 h were performed in combination with the above physical characterizations. As shown in Supplementary Fig. 12b, XRD results indicate the absence of patterns corresponding to crystalline Cu from the initial 10 minutes of the reaction, a trend that persists consistently over the course of 10 hours. This indicates a rapid formation of the amorphous Cu layer under COR conditions, and the amorphous Cu layer remains relatively stable over an extended duration. Through ex situ STEM analysis, we further confirm the presence of an outer layer of amorphous Cu on top of the crystalline Ag nanoparticles. As depicted in Supplementary Fig. 12a, following COR for the durations of 1, 5, and 10 h, Cu amorphous layers with an approximate width of 2–3 nm are always observed at the periphery of rod-shaped particles, consistent with the phenomenon observed in XRD. However, due to the immiscibility of Cu and Ag, we hypothesize that the phase separation between Cu and Ag will become more pronounced with longer reaction times, potentially weakening the influence of Ag on the structural evolution of Cu. Hence, we do not exclude the possibility of crystalline Cu particle formation after prolonged COR (vide infra). Moreover, the *operando* XAFS collected at different COR durations clearly demonstrate the reduction of the long-range ordered structure of Cu with the increase of Ag content. As shown in Supplementary Fig. 12d, after COR on Cu_3_Ag_7_ samples for 1, 5, and 10 h, the structural integrity of Cu in Cu_3_Ag_7_ is essentially maintained, and the long-range order of Cu after 5 and 10 h of COR remains substantially lower than that of the Cu foil, suggesting that the low Cu crystallinity can be maintained under COR conditions for prolonged reaction times. Finally, COR products were also collected and quantified to demonstrate the robust COR activity and selectivity during these COR assessments (Supplementary Fig. 12e).

### Catalyst structure/performance correlations

**Fig. 4:**
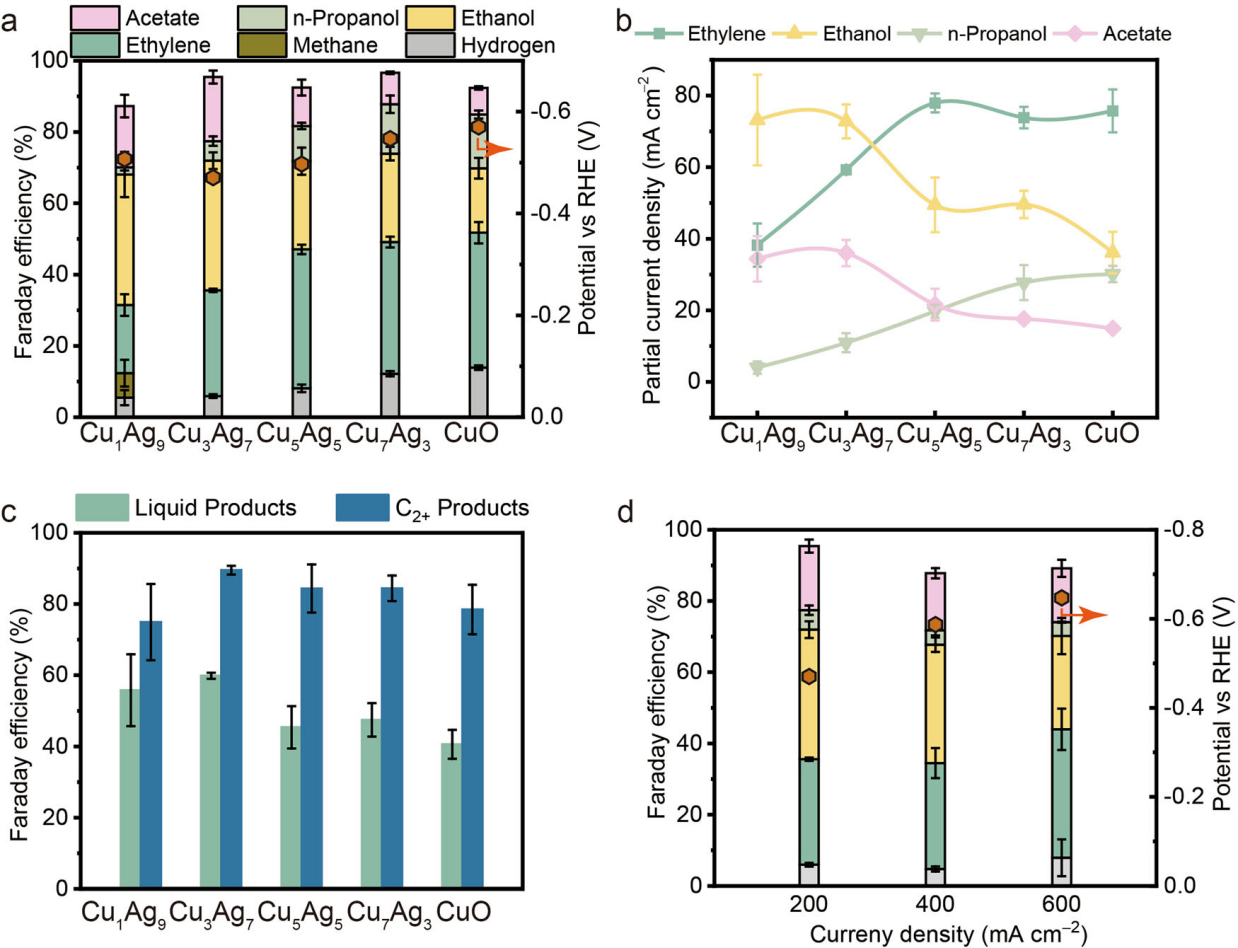
Electrochemical COR performance of CuO and the CuAg catalysts. **a** FEs of hydrogen, methane, ethylene, acetate, ethanol and propanol during COR at 200 mA cm^−2^ on CuO and different CuAg catalysts. **b** Partial current densities of C_2+_ products for CuO and the CuAg catalysts with different Cu/Ag ratio at 200 mA cm^−2^. **c** Total FEs of C_2+_ products and liquid products at 200 mA cm^−2^ on CuO and CuAg samples. **d** FEs of each product on Cu_3_Ag_7_ under different current densities and electrode potentials. The error bars represent standard deviations from at least three independent measurements. Relevant source data are provided as a Source Data file.

The electrochemical COR performance of CuAg and CuO were assessed using a gas diffusion electrode-based flow cell with 1.0 M KOH as the electrolyte. The gaseous and liquid products were detected and quantified by gas chromatography (GC) and Nuclear Magnetic Resonance (NMR) spectroscopy, respectively. We first conducted COR on a pristine Ag catalyst under the identical conditions (Supplementary Fig. 15), where H_2_ was observed as the only product, suggesting that Ag is inert in further reducing CO, at least under our testing conditions. Figure 4a presents the product distributions of COR on CuO and on CuAg with various Cu/Ag ratio at 200 mA cm^−2^. First, the CuAg bimetallic materials demonstrate slightly improved selectivity towards C_2+_ products in comparison to the pristine CuO sample. This improvement is predominantly due to the suppressed H_2_ production, notably pronounced for the CuAg catalyst with high Ag content (Supplementary Fig. 16). As shown in Fig. 4a, despite the marginal enhancement on the C_2+_ selectivity, the CuAg catalysts, i.e., Cu_5_Ag_5_ and Cu_3_Ag_7_, exhibit substantially reduced overpotentials (~0.1 V) at 200 mA cm^−2^ compared to those of the pristine CuO. Given their comparable electrochemical active surface areas (ECSA, Supplementary Fig. 17), we believe that these CuAg bimetallic catalysts show enhanced intrinsic activity towards CO reduction. Specifically, the Cu_3_Ag_7_ exhibits the highest selectivity towards C_2+_ products, with over 60% being liquid products, i.e., ethanol, acetate, and small portions of propanol. However, when the Ag content further increases (Cu_1_Ag_9_), the generation of methane becomes notable on the expense of C_2+_ selectivity. This is possibly due to the insufficient Cu-active sites, leading to the increased COR concentration overpotential (Fig. 4a). We further compared the partial current densities of each C_2+_ products on these CuAg catalysts. The results, depicted in Fig. 4b, reveal that with an increase in the Ag content, the production distribution shifts from gaseous product (ethylene) to liquid products (mainly ethanol and acetate). Worth noting, the formation of n-propanol also decreases along with ethylene upon the addition of Ag, possibly indicating a potential shared pathway and/or intermediates between these two products. Similar hypothesis was made elsewhere recently^78^. Furthermore, taking Cu_3_Ag_7_ as an example, its high selectivity towards C_2+_ products is sustained even under higher current densities (Fig. 4d), with only negligible increase in the H_2_ selectivity, showing its promise for future implementations. Overall, the selectivity towards liquid products is enhanced in the CuAg samples (Fig. 4c), along with an improvement in combined C_2+_ products primarily due to the suppressed H_2_ production.

As discussed above, the substantial Ag content with the bimetallic materials fosters the formation of amorphous Cu layers characterized by below-average coordination numbers during COR. Therefore, we believe that this undercoordinated amorphous Cu prompts overall activity toward C_2+_ formation activity and meanwhile favors the production of liquid products^79,80^. To validate our hypothesis, we conducted COR on a physical mixture of CuO and Ag_2_O with the same atomic ratio as the CuAg catalysts. Under the same conditions, although the physically mixed Cu/Ag catalyst also exhibit reduced formation of H_2_ with the increase of Ag content (Supplementary Fig. 18), the selectivity towards ethylene and liquid products resemble that of the pristine CuO catalyst. Hence, we can infer that the enhanced C_2+_ formation activity and improved liquid product selectivity is predominately resulted from the amorphous nature of the Cu. Note, our conclusion does not rule out the possibilities proposed by the recent studies on COR using other CuAg bimetallic materials, the variations are likely resulted from differences in the synthetic procedures employed for preparing the catalyst materials^32,33,37,42^. For instance, the active sites suggested to be in close proximity to the Cu and Ag interface could also contribute to our scenario since the Cu and Ag are phase-separated^44^.

**Fig. 5:**
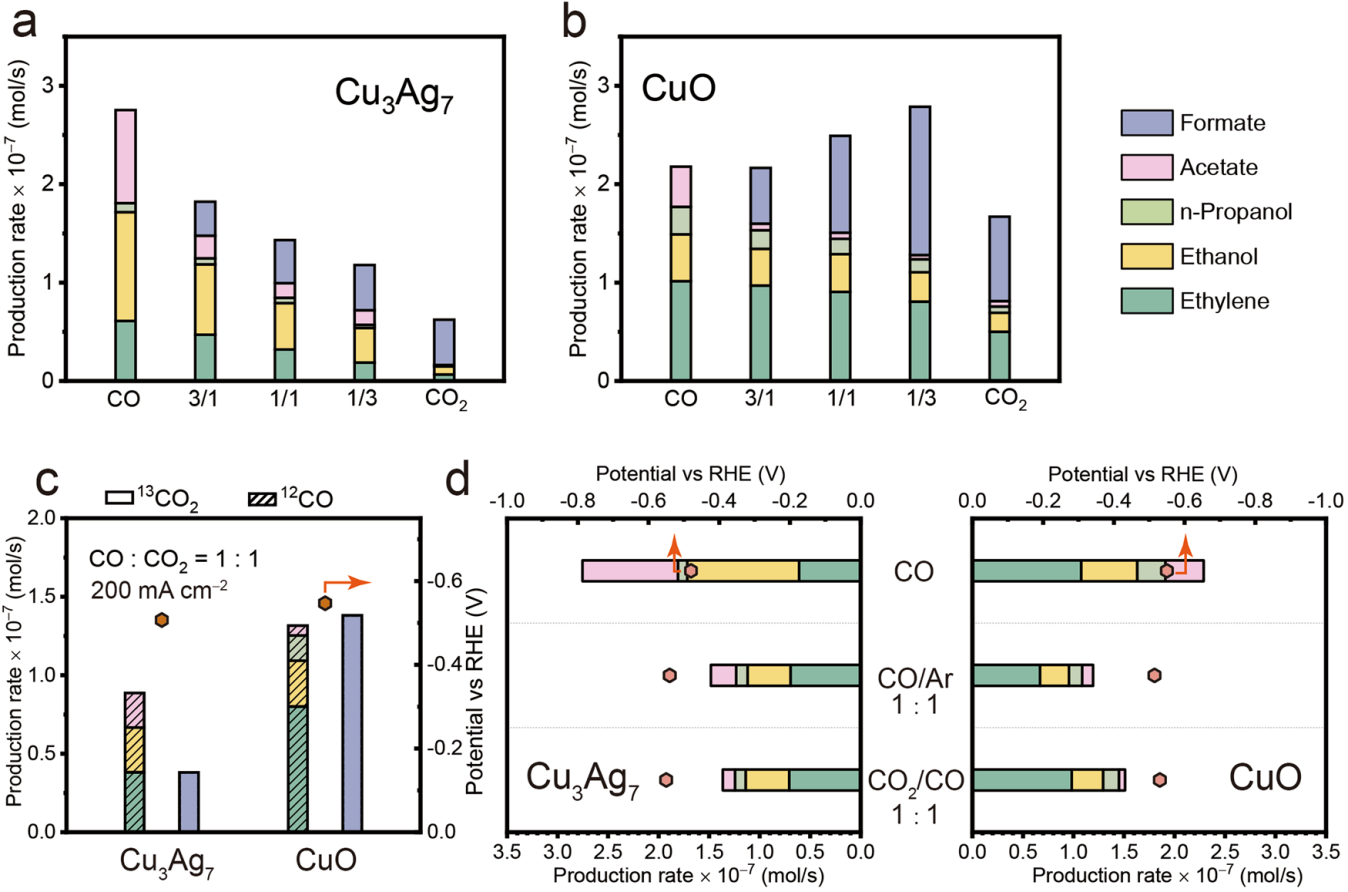
CO/CO_2_ co-feeding electrolysis and isotopic labeling experiments. Production rates of hydrogen, carbon monoxide, ethylene, acetate, ethanol and propanol on **a** Cu_3_Ag_7_ and **b** CuO catalysts across various feed-gas ratios under 200 mA cm^−2^. **c** Product distribution on Cu_3_Ag_7_ and CuO catalysts using isotopic labeled stream of ^13^CO_2_/^12^CO under 200 mA cm^−2^. **d** C_2+_ products distribution on CuO and Cu_3_Ag_7_ catalysts across various feed-gas ratios. Relevant source data are provided as a Source Data file.

Previously, it was demonstrated that electrolysis involving the co-feeding of CO/CO_2_ promotes the C_2+_ product formation during COR^44,81,82^. Inspired by this, we explored whether this phenomenon could be extended in flow cells with higher current densities. Consequently, we carried out similar CO/CO_2_ co-feeding electrolysis in flow cell at 200 mA cm^−2^ (Fig. 5a, b). We also evaluated the electrocatalytic performance of the CuAg catalysts in pure CO_2_ (CO_2_R) for comparative analysis (Supplementary Fig. 19). Under the typical CO_2_R condition, instead of C_2+_ products, CO emerges as the primary product, particularly evident on catalysts with high Ag content such as Cu_3_Ag_7_. This result is anticipated since Ag is known as an active and selective catalyst for the CO_2_ to CO conversion. Upon mixing CO with CO_2_, depicted in Fig. 5a, b, both Cu_3_Ag_7_ and CuO exhibit enhanced formation rate for C_2+_ products. This phenomenon is more profound on Cu_3_Ag_7_, which is likely due to its improved activity for CO reduction as aforementioned. For instance, the primary product on Cu_3_Ag_7_ is CO with a rate of ~9.67 × 10^−7^ moles s^−1^ using pure CO_2_ feeding at 200 mA cm^−2^, and the production rate of C_2+_ is only 1.62 × 10^−8^ moles s^−1^. Notably, upon introducing 25% CO into the feed stream, the production rate of C_2+_ products increase to 7.19 × 10^−8^ moles s^−1^, which accounts for fivefold increase in comparison to pure CO_2_R. However, this increasing trend in C_2+_ formation did not decline similarly to the previous studies^44,81^ when using only CO as the feed, on both Cu_3_Ag_7_ and CuO. At first glance, this phenomenon suggests that CO_2_R may compete with COR for active sites, and COR pathways are inherently more active for C_2+_ production, within vaper fed devices. This result aligns well with a recent report, which highlighted the significance of conducting electrolysis under high CO concentrations^83^. To validate this hypothesis, we firstly conduct identical co-feeding electrolysis using isotopically labeled ^12^CO/^13^CO_2_ stream in 1 M KOH. Surprisingly, as shown in Fig. 5c, all measurable C_2+_ products are originated from the ^12^CO on both Cu_3_Ag_7_ and CuO, indicating that they are derived from the COR pathway exclusively. Similar, though not identical, observations were made in a recent work^44^, where the majority of C_2+_ products also originated from the COR pathway. The authors attribute this behavior to the hypothesis of Cu having distinct reaction sites on polycrystalline Cu, favoring the conversion of CO_2_ to CO (Cu_CO2_) and the reduction of CO to C_2+_ (Cu_CO_) products, respectively. We believe that in our case, the flow cell configuration provides a higher concentration of CO, which is expected to bind to the surface more readily, thereby saturating the Cu-active sites for C_2+_ production. Nevertheless, to provide additional evidence that CO_2_ does not contribute to the formation of C_2+_ products under our testing conditions, we carried out Argon (Ar)/CO co-feeding electrolysis (Fig. 5d). Under similar applied overpotentials, the production rate of C_2+_ products on both Cu_3_Ag_7_ and CuO with Ar/CO feeding and CO_2_/CO feeding is comparable, indicating that CO predominately contributes to the generation of C_2+_ products.

However, it is anticipated that not all CO_2_ molecules participate in the electrochemical reaction due to the reaction between CO_2_ and KOH. To investigate this effect further, we conducted identical CO/CO_2_ co-feeding electrolysis using isotopically labeled ^13^CO_2_ in electrolytes with varying pH, such as KHCO_3_ and H_2_SO_4_. As shown in Supplementary Fig. 21b, c, upon mixing ^12^CO with ^13^CO_2_, Cu_3_Ag_7_ demonstrates an enhanced formation rate for C_2+_ products in both 1 M KHCO_3_ and 0.1 M H_2_SO_4_. The increasing trend of C_2+_ products remains consistent across different electrolytes when the CO content is increased, pinpointing the contribution of CO to the generation of C_2+_ products, as discussed previously. Note, despite the overall similar trends in product distributions, there are minor differences exists among different experiments, likely attributable to slight variations in the applied potentials in different systems. In all experiments, C_2+_ products are predominantly formed solely by ^12^C-atoms originating from ^12^CO, with a minor contribution from ^13^CO_2_ (Supplementary Fig. 21f). For instance, over 80% of the ethanol and acetate produced originated solely from ^12^CO. Even in the remaining ~20% of the ethanol and acetate, ^13^C contributes only one carbon, specifically the methyl group in both products. Additionally. there are no C_2+_ products formed solely by ^13^C-atoms from ^13^CO_2_. Therefore, we believe that ethanol and acetate are formed through the pathway of ^12^CO–^13^COH asymmetric coupling^84^. Nevertheless, these results further support our previous conclusion that the majority of C_2+_ products originated from the COR pathway under our testing conditions. It is important to note that an increase in local pH is inevitable even though acidic electrolytes are employed, therefore, we do not exclude the contribution of the reaction between a portion of CO_2_ and KOH, thereby diminishing the contribution of CO_2_ to C_2+_.

We also analyzed the production rate of formate during the co-feeding electrolysis. Notably, as shown in Fig. 5b, we observed an increase in formate production rate when blending the CO_2_ stream with 25% CO, instead of decline. Since formate is expected to be formed exclusively from CO_2_, we suppose that it is generated on the same type of active site as CO formation (Cu_CO2_), and the external CO feeding shifts the chemical equilibrium towards formation production. Nonetheless, as we further increase the CO concentration in the feed, the insufficient CO_2_ partial pressure eventually leads to the decline in formate production (Fig. 5b). On the other hand, Cu_3_Ag_7_ exhibits substantially reduced production rate for formate, which is also relatively insensitive to the CO_2_ participial pressure. We attribute this to the undercoordinated nature of the amorphous Cu layer on Cu_3_Ag_7_, which is consistent with the hypothesis proposed in previous work. It is likely that the Cu_CO2_ sites associated with the close-packed facets, such as Cu (111). Nevertheless, as shown in Fig. 5a, b, we can conclude that the rates of CO_2_ to formate conversion are comparable, if not higher, than those of C_2+_ formation. The introduction of Ag effectively suppresses the competing pathway for producing multi-carbon products.

### Theoretical investigations for COR on amorphous Cu

**Fig. 6:**
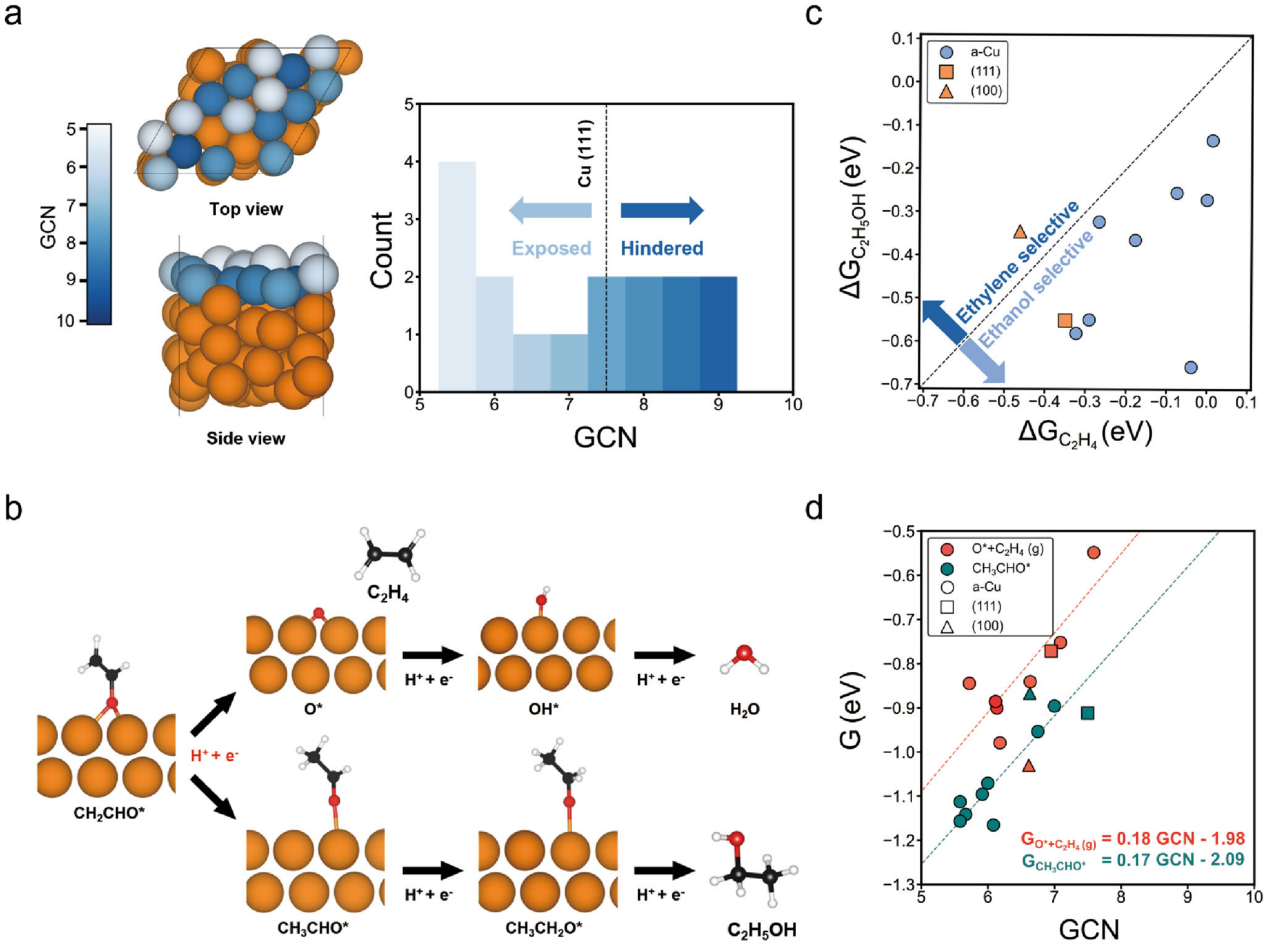
DFT calculations on Cu and amorphous Cu. **a** Top and side views of amorphous Cu (denoted as a-Cu), where darker colors indicate higher generalized coordination number (GCN) values. The histogram displays GCN in the exposed and hindered sites of the first layer. The dash line indicates the GCN calculated for an ideal Cu (111) surface. **b** Reaction pathway for ethylene and ethanol production by COR. The orange, red, black and white spheres represent the Cu, O, C, and H atoms, respectively. **c** The Gibbs free energy changes of ethylene-selective pathway ($\triangle {{{{{{\rm{G}}}}}}}_{{{{{{{\rm{C}}}}}}}_{2}{{{{{{\rm{H}}}}}}}_{4}}$) and ethanol-selective pathway ($\triangle {{{{{{\rm{G}}}}}}}_{{{{{{{\rm{C}}}}}}}_{2}{{{{{{\rm{H}}}}}}}_{5}{{{{{\rm{OH}}}}}}}$) on a-Cu illustrating the selectivity between two products. **d** The binding free energies of CH_3_CHO* (green) and O* + C_2_H_4_ (g) (red) vs. GCN values of the active sites of a-Cu. The dashed line in panel d represents the observed correlation between the GCN of active sites and the binding free energies. Relevant source data are provided as a Source Data file.

Density functional theory (DFT) based calculations were conducted to understand the improved selectivity towards liquid oxygenates such as ethanol on amorphous Cu. An amorphous Cu structure (a-Cu) was constructed using ab initio molecular dynamics (AIMD) simulations, where the details can be found in the Method section and Supplementary note 1. As shown in Fig. 6a, the a-Cu structure displays a roughened surface layer compared to the crystalline Cu (111) facet. For the outermost Cu atoms on the a-Cu surface layer, we characterized these sites with the generalized coordination number (GCN), an effective geometry descriptor that reflects the coordination environments of the Cu atoms (Supplementary Note 2 and Fig. 6a). Clearly, the Cu atoms exposed on the surface exhibit lower GCN values in comparison to the more hindered sites, all of which are lower than 7.5, the GCN value of an ideal Cu (111) surface atom. This result is in accordance with our EXAFS spectra, implying the reduction of the coordination number of the Cu (Fig. 3d). Considering the correlation between GCN values and binding energies, we anticipate that the a-Cu structure can enhance the reactivity of the surface Cu atoms (Supplementary Fig. 23)^79^.

Previously, it has been proposed that the product selectivity between ethylene and ethanol depends on the energetics of reaction pathways involving the intermediate of CH_2_CHO* (Fig. 6b)^84,85^. Specifically, ethylene is produced when the C atom in the CHO moiety within CH_2_CHO* is protonated, leading to the breaking of the C–O bond (CH_2_CHO* → O* + C_2_H_4_ (g)). On the other hand, the production of ethanol occurs when C atom in the CH_2_ within CH_2_CHO* undergoes protonation, followed by the consequent electron coupled protonation steps (CH_2_CHO* → CH_3_CHO* → C_2_H_5_OH (g)). Thus, we calculated the Gibbs free energy changes of pathways leading to ethylene and ethanol, denoted as $\Delta {{{{{{\rm{G}}}}}}}_{{{{{{{\rm{C}}}}}}}_{2}{{{{{{\rm{H}}}}}}}_{4}}$ and $\Delta {{{{{{\rm{G}}}}}}}_{{{{{{{\rm{C}}}}}}}_{2}{{{{{{\rm{H}}}}}}}_{5}{{{{{\rm{OH}}}}}}}$, respectively, and compared them across various adsorption sites on the a-Cu surface. Specifically, we calculated ${\Delta {{{{{\rm{G}}}}}}}_{{{{{{{\rm{C}}}}}}}_{2}{{{{{{\rm{H}}}}}}}_{4}}$ and ${\Delta {{{{{\rm{G}}}}}}}_{{{{{{{\rm{C}}}}}}}_{2}{{{{{{\rm{H}}}}}}}_{5}{{{{{\rm{OH}}}}}}}$ as follows:

1 $${\Delta {{{{{\rm{G}}}}}}}_{{{{{{{\rm{C}}}}}}}_{2}{{{{{{\rm{H}}}}}}}_{4}}=\,{{{{{{\rm{G}}}}}}}_{{{{{{\rm{O}}}}}} \ast+{{{{{{\rm{C}}}}}}}_{2}{{{{{{\rm{H}}}}}}}_{4}({{{{{\rm{g}}}}}})}-{{{{{\rm{G}}}}}}_{{{{{\rm{C}}}}}{{{{{\rm{H}}}}}}_{2}{{{{\rm{CHO}}}}}*}$$

2 $${\Delta G}_{{C}_{2}{H}_{5}{OH}}=\,{{{{{\rm{G}}}}}}_{{{{{\rm{C}}}}}{{{{{\rm{H}}}}}}_{3}{{{{\rm{CHO}}}}}*}-{{{{{\rm{G}}}}}}_{{{{{\rm{C}}}}}{{{{{\rm{H}}}}}}_{2}{{{{\rm{CHO}}}}}*}$$

For all distinct exposed and hindered sites, we calculated and compared $\Delta {{{{{{\rm{G}}}}}}}_{{{{{{{\rm{C}}}}}}}_{2}{{{{{{\rm{H}}}}}}}_{4}}$ and $\Delta {{{{{{\rm{G}}}}}}}_{{{{{{{\rm{C}}}}}}}_{2}{{{{{{\rm{H}}}}}}}_{5}{{{{{\rm{OH}}}}}}}$, where more energetically favorable $\Delta {{{{{{\rm{G}}}}}}}_{{{{{{{\rm{C}}}}}}}_{2}{{{{{{\rm{H}}}}}}}_{4}}$ ($\Delta {{{{{{\rm{G}}}}}}}_{{{{{{{\rm{C}}}}}}}_{2}{{{{{{\rm{H}}}}}}}_{5}{{{{{\rm{OH}}}}}}}$) corresponds to selective production of ethylene (ethanol). We found that geometric relaxations of many hindered sites resulted in the relocation of adsorbates to the exposed sites, suggesting that the exposed sites are the predominate active sites on a-Cu. Therefore, only the exposed sites were considered for further calculations. As shown in Fig. 6c, we observed that all adsorption sites of a-Cu exhibited a more favorable $\Delta {{{{{{\rm{G}}}}}}}_{{{{{{{\rm{C}}}}}}}_{2}{{{{{{\rm{H}}}}}}}_{5}{{{{{\rm{OH}}}}}}}$, suggesting a general preference for ethanol formation over ethylene.

To gain deeper understandings of the enhanced ethanol selectivity on the a-Cu, we investigated the energetics of O* and CH_3_CHO* in detail, as their binding strengths play critical roles in determining the selectivity^86,87^. As shown in Fig. 6d, we observed strong correlations between the GCN of the active sites and the binding free energies of both O* and CH_3_CHO*. Notably, while their slopes were found to be similar, ${{{{{\rm{G}}}}}}_{{{{{\rm{C}}}}}{{{{{\rm{H}}}}}}_{3}{{{{\rm{CHO}}}}}*}$ exhibited lower y-intercept than ${{{{{\rm{G}}}}}}_{{{{{\rm{O}}}}} \ast+{{{{{\rm{C}}}}}}_{2}{{{{{\rm{H}}}}}}_{4}({{{{\rm{g}}}}})}.$ This implies that the ethanol pathway is generally more favorable than the ethylene pathway on active sites with the same GCN, with the exception of the Cu(100) sites. Furthermore, for a-Cu, O* adsorption mostly exhibits a higher GCN compared to CH_3_CHO* adsorption due to O* preferring many-fold sites such as bridge or hollow sites, while CH_3_CHO* prefers low-fold sites such as top or bridge sites. We also note that the GCN changes by less than 1 for various adsorption sites on crystalline Cu (111) and (100) facets (Supplementary Table 7), while it changes by more than 4 for a-Cu. This drastic difference results in a more significant stabilization of CH_3_CHO* on a-Cu, consequently, leading to the selective production of ethanol. Overall, the computational simulations confirm that the improved selectivity and activity towards ethanol production on a-Cu originate from the amorphization of Cu, which reduces the coordination of surface atoms by roughening the surface.

## Discussion

In this work, we interrogated the structural evolution of a typical Cu/Ag oxide-derived bimetallic catalyst during COR under practical relevant testing conditions. Through comprehensive in situ and ex situ characterizations, we found that the homogenous Cu/Ag oxide precursors undergo a structural transformation, resulting in a bimetallic composite comprising small Ag nanoparticles enveloped by thin and amorphous Cu layers. These amorphous Cu layers exhibit substantially reduced average coordination numbers compared to the polycrystalline Cu. As a result, this bimetallic catalyst demonstrates improved activity and high selectivity towards C_2+_ products (>90%), particularly liquid products (>60%), at high current densities under typical COR testing conditions. During CO_2_/CO co-feeding electrolysis in the flow cell configuration, we found that a high CO concentration is more favorable for targeting C_2+_ product production. In addition, introducing Ag to Cu can suppress the competing pathway for formate production in the presence of CO_2_. Furthermore, our DFT-based calculations further confirmed the low coordination nature of the amorphous Cu sites, and explained their high selectivity towards liquid products like ethanol, which is due to the favorable protonation of the C atom in the CH_2_ moiety within the intermediate of CH_2_CHO*. Overall, we believe our research can guide investigations into catalyst dynamic structural evolutions during electrochemical CO_2_/CO reduction, offering insights for future catalyst and catalytic system design.

## Methods

### Chemicals and materials

Copper (II) nitrate trihydrate (Cu (NO_3_)_2_ · 3H_2_O, 99.7%, trace metals basis), Silver nitrate (AgNO_3_, ACS reagent, ≥99%), Sodium hydroxide (NaOH, reagent grade, ≥97%), Ethyl alcohol (C_2_H_6_O, ≥99%) were used as purchased from Sigma Aldrich. Potassium Hydroxide (KOH, 99.99%) was used as purchased from Macklin, Polytetrafluoroethylene (PTFE, 60 wt % dispersion in H_2_O), Nafion™ perfluorinated resin solution (5 wt. % in mixture of lower aliphatic alcohols and water, contains 45% water) were used as purchased from Sigma Aldrich. The carbon paper (YLS-30T) was purchased from Su Zhou Sinero Technology Co., LTD. Anion exchange membrane (AEM, Selemion AMN/N type 1, AGC Inc.) was used to separate working and counter electrodes. CO and CO_2_ were purchased from Air liquid. All chemicals are commercially available without further treatment. Ultra-pure water (18.2 MΩ. Cm, Millipore) was used throughout the experiments.

### Synthesis of CuAg, CuO, and Ag_2_O precursors

The CuAg precursors were synthesized as follows. First, Cu (NO_3_)_2_ and AgNO_3_ with designed atomic ratio were dissolved in 10 mL H_2_O. In total, 20 mL of sodium hydroxide solution containing 2.4 g NaOH was added into above solution and the brown precipitate formed instantly. After stirring for 1 h, the precipitate was transferred into 50-mL centrifuge tube and was centrifuged at 4830 × *g* for 5 min. The product was washed with water. After repeating this process for five times, the product was dried at 100 °C in air for 12 h. The CuO and Ag_2_O precursors were prepared by the same process with Cu (NO_3_)_2_ and AgNO_3_, respectively.

### Material characterizations

The surface morphology of samples was characterized by JEOL JSM-7610F SEM. Transmission electron microscopy (TEM) image and X-ray energy-dispersive spectroscopy (XEDS) were obtained by a JEOL JEM-2010F TEM. X-ray diffraction (XRD, Bruker D8-advance) was applied to characterize the crystal structure with a Cu K-α as X-ray source n (*λ* = 1.5406 Å). X-ray photoelectron spectroscopy (XPS) was conducted to analysis the surface chemical compositions on Kratos Axis Ultra spectrometer (Mono Al Kα, hν = 1486.71 eV). High-angle annular dark-field scanning transmission electron microscopy (HAADF-STEM) and energy-dispersive X-ray spectroscopy were conducted on a JEOL ARM 200CF equipped with an Oxford Instruments X-ray energy-dispersive spectrometer.

### Operando measurements

*Operando* X-ray absorption fine structure (XAFS) spectroscopy was performed at the XAFCA beamline of the Singapore Synchrotron Light Source in the fluorescence mode. A customized flow cell with alkaline (1 M KOH) electrolyte for COR was used. A Kapton tape was used to seal the gas chamber at the cathode side to allow the penetration of X-ray and fluorescence signals. Cu K-edge XAFS spectra were measured with electron energy of 0.7 GeV. X-ray absorption near-edge spectra (XANES) and extended X-ray absorption fine structure (EXAFS) spectra were analyzed by using Athena and Artemis included in the Demeter package. The standard reference material of Cu foil was measured in parallel. The energy was calibrated with Cu foil for Cu K-edge. Electrochemical tests were conducted with a CHI760E potentiostat. Cu K-edge XANES and EXAFS spectra were collected. All characterization were conducted at room temperature (~25 °C) and ambient pressure.

### Preparation of gas diffusion electrode

The catalyst was sprayed on the carbon paper with 1 × 1 cm^−2^. In all, 1 mg CuO/CuAg precursors powder was dispersed in the mixed solution with 5 wt% Nafion solution and 1 ml of ethanol. The ratio between Nafion and catalysts powder is 4 μl Nafion/1 mg catalyst powder. The ink was dispersed in an ultrasonic machine for more than 5 min before being sprayed on the carbon paper. After the ink is prepared, the carbon paper is fixed on the heating plate. The ink was added into the spray gun to spray on the carbon paper with 1 mg/cm^2^ to form catalyst film. Flow cell measurements were conducted in a self-made cell (1 cm^2^ active area). This cell was assembled from the sequential stacking of a gas chamber, a catalyst-loaded gas diffusion electrode (GDE) cathode, a catholyte chamber (where an Ag/AgCl reference electrode locates), an anion exchange membrane, an anolyte chamber and an IrO_2_-loaded Ti plate anode. The Ag/AgCl reference electrode was purchased from Gaoss Union (Model 1038) and used after calibration using a homemade standard hydrogen electrode. The IrO/Ti anode was prepared by etching the Ti mesh with boiling 6 M HCl (ACS reagent, 37%, Sigma Aldrich) for 40 min. Then the solution with 2 mL HCl, 18 mL isopropanol and 60 mg Iridium chloride hydrate (99.9%, Sigma Aldrich) was dip-coated on Ti mesh, followed by drying under 100 °C for 10 min and calcined in air at 500 °C for 10 min. The procedure was repeated ten times. During the typical COR measurements, 22 sccm of CO was continuously fed to the gas chamber. The catholyte electrolyte (1 M KOH) was pumped to circulate through the catholyte chambers and anolyte chambers (1 M KOH) at the rate of 15 mL/min by a double-channel peristaltic pump. The pH of the electrolyte was measured using a benchtop pH Meter (Fisher Scientific AE150 Accumet).

### Feed gases controlling

The flow rate is kept at 22 sccm constantly. With the increased amount of CO co-feeding, the feed-gas ratio is tuned from 3:1 (CO_2_ to CO), 1:1 to 1:3. Accordingly, the concentration of CO_2_ or CO in solution was dependent on the partial pressure of the gases in the atmosphere (298 K, 101.3 KPa).

### Electrochemical measurements

A BioLogic VMP3 multichannel potentiostat/galvanostat with a built-in EIS analyzer was used for all the electrochemical measurements under ambient conditions (room temperature around 25 °C) and ambient pressure. The anion exchange membrane (Selemion AMN/N type 1, AGG Inc.) was used between the working and counter electrode compartments. CO was flowed through the working-electrode compartment, with the flow rate regulated by a mass flow controller at 22 sccm. All the electrochemical performances shown in this work were tested for 0.5 h. The potential readings were measured against Ag/AgCl and then converted to RHE with necessary *i*R compensation by equation:

3 $${{{{{\rm{E}}}}}}({{{{{\rm{vs}}}}}}.{{{{{\rm{RHE}}}}}}) \,=\,{{{{{\rm{E}}}}}}({{{{{\rm{vs}}}}}}.{{{{{\rm{Ag}}}}}}/{{{{{\rm{AgCl}}}}}}) \,+\,0.197{{{{{\rm{V}}}}}}+0.0591{{{{{\rm{V}}}}}}\,\times \,{{{{{\rm{pH}}}}}}-{{{{{\rm{iR}}}}}}$$

The solution resistances were tested using the impedance module in potentiostat (BioLogic VMP3) during each electrochemical measurement, and the corresponding solution pH was measured using a benchtop pH Meter (Fisher Scientific AE150 Accumet). The Faradic Efficiency (FE) of each product was calculated by following equation:

4 $${{{{{\rm{FE}}}}}}\left(\%\right) \,=\,\frac{{{{{{\mathrm{amount}}}}}} \,{{{{{\mathrm{of}}}}}} \,{{{{{\mathrm{the}}}}}} \,{{{{{\mathrm{product}}}}}}\,({{{{{\mathrm{mol}}}}}})\times n\times F\left(\frac{C}{{mol}}\right)}{I\left(A\right)\times t\,\left(s\right)}\times 100$$

where *n* represents the number of electrons transferred, *F* represents the Faradaic constant, and *C* represents the Coulomb number.

### Product analysis

For each tested cathode potential, 1 ml of reactor exhaust gas was injected into a gas chromatograph (GC, Shimadzu 2014). Liquid products were measured by ^1^H NMR spectrum (Bruker, 400 MHZ system) with Phenol and DMSO as the internal standard. A 700 μL aliquot of post-reaction electrolyte was mixed with 35 μL of the internal standard (10 mM dimethyl sulfoxide and 50 mM phenol in D_2_O solution) for quantification.

### Computational details

Density functional theory (DFT) calculations were performed using Vienna Ab initio Simulation Package (version 5.4.4). The generalized gradient approximation with the revised Perdew-Burke-Ernzerhof (GGA-RPBE) functional was used to describe the exchange-correlation interactions. The cutoff energy was set to 500 eV, and the convergence tolerances of energy and force were set to 10^−4^ eV and 0.05 eV/Å, respectively. The Monkhorst-Pack *k-*point meshes were set to (2$\times$2$\times$1). A four-layered ($4\times$4) supercell consisting of 64 atoms was used to model crystalline and amorphous Cu (111). Further details on the construction of amorphous Cu (a-Cu) can be found in the Supplementary note 1. The bottom two layers of the structures were fixed to their bulk positions during the relaxation. A vacuum layer of ~15 Å along the z-direction was added.

We used the computational hydrogen electrode (CHE) method to include the effect of the applied potential. This method assumes an equivalent chemical potential for half the amount of H_2_ gas and a proton-electron pair ($\mu$(H^+^ + e^-^) = 0.5$\mu$(H_2_)) under standard conditions (pH = 0 and ${{{{{\rm{P}}}}}}_{{{{{{{\rm{H}}}}}}}_{2}}$ = 101,325 Pa) in the absence of the applied potential (*U* = 0 V_RHE_). The Gibbs free energies were calculated by adding the Gibbs free energy correction values (${{{{{{\rm{G}}}}}}}_{{{{{{\rm{corr}}}}}}}$) to DFT energies (E_DFT_), i.e., ${{{{\rm{G}}}}}=\,{{{{{\rm{E}}}}}}_{{{{{\rm{DFT}}}}}}+{G}_{{corr}}$. The Gibbs free energy correction values of adsorbates (gaseous molecules) were calculated using the harmonic oscillator (ideal gas) approximation at 298.15 K as implemented in the Atomic Simulation Environment (ASE). We included the solvation effect using the implicit solvation method implemented as VaspSol. To correct DFT errors of gas-phase molecules originated from RPBE functional, we added +0.07 eV and +0.03 eV to the DFT energy of CO and C_2_H_4_ molecule, respectively.

The binding free energies of the reaction intermediates were calculated relative to gas-phase CO, H_2_, and H_2_O as follows:

5 $${{{{{\rm{G}}}}}}_{{{{{{\rm{CH}}}}}}_{2}{{{{\rm{CHO}}}}}*}={{{{{\rm{E}}}}}}_{{{{{{\rm{CH}}}}}}_{3}{{{{\rm{CHO}}}}}*}-{{{{{\rm{E}}}}}}_{*}-{2{{{{\rm{E}}}}}}_{{{{{\rm{CO}}}}}}-{2.5{{{{\rm{E}}}}}}_{{{{{{\rm{H}}}}}}_{2}}+{{{{{\rm{E}}}}}}_{{{{{{\rm{H}}}}}}_{2}{{{{\rm{O}}}}}}+{G}_{{corr}}$$

6 $${{{{{\rm{G}}}}}}_{{{{{{\rm{CH}}}}}}_{3}{{{{\rm{CHO}}}}}*}={{{{{\rm{E}}}}}}_{{{{{{\rm{CH}}}}}}_{3}{{{{\rm{CHO}}}}}*}-{{{{{\rm{E}}}}}}_{*}-{2{{{{\rm{E}}}}}}_{{{{{\rm{CO}}}}}}-{3{{{{\rm{E}}}}}}_{{{{{{\rm{H}}}}}}_{2}}+{{{{{\rm{E}}}}}}_{{{{{{\rm{H}}}}}}_{2}{{{{\rm{O}}}}}}+{G}_{{corr}}$$

7 $${{{{{\rm{G}}}}}}_{{{{{{\rm{O}}}}} \ast+{{{{\rm{C}}}}}}_{2}{{{{{\rm{H}}}}}}_{4}({{{{\rm{g}}}}})}={{{{{\rm{E}}}}}}_{{{{{\rm{O}}}}}*}-{{{{{\rm{E}}}}}}_{*}-{2{{{{\rm{E}}}}}}_{{{{{\rm{CO}}}}}}-{3{{{{\rm{E}}}}}}_{{{{{{\rm{H}}}}}}_{2}}+{{{{{\rm{E}}}}}}_{{{{{{\rm{H}}}}}}_{2}{{{{\rm{O}}}}}}+{{{{{\rm{E}}}}}}_{{{{{{\rm{C}}}}}}_{2}{{{{{\rm{H}}}}}}_{4}}+{G}_{{corr}}$$

### Supplementary information

Supplementary Information

Peer Review File

### Source data

Source data

## Data Availability

Source data are provided with this paper and are available from the corresponding authors upon request.
