# Supplementary material for: Revealing the structural evolution of CuAg composites during electrochemical carbon monoxide reduction
Source: Nat Commun. 2024 Jun 1;15:4692. doi: 10.1038/s41467-024-49158-4 (PMC11144262; doi:10.1038/s41467-024-49158-4)
Supplement: Supplementary file 1 — Supplementary Information [file 41467_2024_49158_MOESM1_ESM.pdf]

Supplementary Information

Revealing the Structural Evolution of CuAg Composites during Electrochemical Carbon Monoxide Reduction

Di Wang,[1, †] Hyun Dong Jung,[2, †] Shikai Liu,[3] Jiayi Chen,[1] Haozhou Yang,[1] Qian He,[3, *] Shibo Xi,[4, *] Seoin Back,[2, *] Lei Wang,[1, 5*]

[1] Department of Chemical and Biomolecular Engineering, College of Design and Engineering, National University of Singapore, 4 Engineering Drive 4, 117585, Singapore

[2] Department of Chemical and Biomolecular Engineering, Institute of Emergent Materials, Sogang University, Seoul 04107, Republic of Korea

[3] Department of Material Science and Engineering, College of Design and Engineering, National University of Singapore, 9 Engineering Drive 1, 117575, Singapore

[4] Institute of Sustainability for Chemicals, Energy and Environment (ISCE2), Agency for Science, Technology and Research (A*STAR), 1 Pesek Road, Jurong Island, 627833, Singapore

[5] Centre for Hydrogen Innovations, National University of Singapore, 1 Engineering Drive 3, 117580, Singapore

[†] These authors contribute to this work equally

Email: heqian@nus.edu.sg, xi_shibo@isce2.a-star.edu.sg, sback@sogang.ac.kr, wanglei8@nus.edu.sg

Content

Supplementary note 1 Construction of Amorphous Cu 4

Supplementary note 2 Generalized Coordination Number (GCN) 4

Supplementary Figures 5

Supplementary Figure 1. Representative SEM images of as-prepared CuO and CuAg samples 5

Supplementary Figure 2. Representative SEM images of post-reacted CuO and CuAg samples 6

Supplementary Figure 3. TEM images of Cu_3Ag_7 samples 6

Supplementary Figure 4. TEM EDS-mapping of post-reacted Cu_5Ag_5 samples 7

Supplementary Figure 5. STEM EDS-mapping of post-reacted Cu_7Ag_3 samples 7

Supplementary Figure 6. Structural characterization of Cu_3Ag_7 catalysts after COR at 200 mA cm^{-2} 8

Supplementary Figure 7. Structural characterization of Cu_3Ag_7 catalysts after COR at 200 mA cm^{-2} 9

Supplementary Figure 8. Model figure of self-designed flow cell for COR/CO_2R 10

Supplementary Figure 9. FT of EXAFS spectra of CuO and CuAg samples at OCV 11

Supplementary Figure 10. *Operando* EXAFS fitting curves 12

Supplementary Figure 11. EXAFS spectra of CuAg samples at –0.65 V vs RHE under COR and Cu foil in (a) k-space and (b) q-space.. 13

Supplementary Figure 12. COR performance and structural information of CuAg samples 14

Supplementary Figure 13. XPS spectra of as-prepared samples 15

Supplementary Figure 14. XPS spectra of Cu LMM spectra of CuO and CuAg samples after COR with 200 mA cm^{-2} for 1h.. 16

Supplementary Figure 15. Electrochemical performance of CO reduction on Ag_2O 17

Supplementary Figure 16. Electrochemical performance of CO reduction.. 18

Supplementary Figure 17. Effective electrochemical active surface area tests (ECSA). 19

Supplementary Figure 18. Electrochemical performance of COR on CuO and physical-mixed CuAg samples at 200 mA cm^{-2}.. 20

Supplementary Figure 19. Electrochemical performance of CO_2 reduction 21

Supplementary Figure 20. Production rates across various feed-gas ratios under 200 mA cm^{-2} 22

Supplementary Figure 21. CO/CO_2 co-feeding electrolysis and isotopic labelling experiments. 23

Supplementary Figure 22. Potential and FEs of ethylene and hydrogen on Cu_5Ag_5 samples in long-time test.......... 24

Supplementary Figure 23. Binding free energies on amorphous Cu and Cu(111) and (100). 25

Supplementary Tables..........26

Supplementary Table 1. ICP-OES analysis of Cu and Ag ratio for CuAg samples. 26

Supplementary Table 2. ICP-OES analysis of Cu and Ag in electrolyte for CuAg samples. 26

Supplementary Table 3. Fitting parameters of Fourier transform (FT) of EXAFS on CuO samples. 26

Supplementary Table 4. Fitting parameters of Fourier transform (FT) of EXAFS on Cu_7Ag_3 samples. 26

Supplementary Table 5. Fitting parameters of Fourier transform (FT) of EXAFS on Cu_5Ag_5 samples. 27

Supplementary Table 6. Fitting parameters of Fourier transform (FT) of EXAFS on Cu_3Ag_7 samples. 27

Supplementary Table 7. GCN of various adsorption sites on Cu facets. 27

Supplementary note 1 Construction of Amorphous Cu

The amorphous Cu (a-Cu) structure was generated through Ab-initio molecular dynamics (AIMD) simulations performed in the NVT ensemble using the Nose-Hoover thermostat and Verlet algorithm. To create the a-Cu structure, a crystalline Cu (4 × 4) supercell was initially heated from 300 K to 5,000 K without any geometric constraints, then held at 5,000 K before being cooled back down to 300 K and maintained at that temperature. Each step of this process lasted for 3 ps, with a time step of 1 fs. The resulting structure obtained at the end of the simulation was used to simulate the a-Cu system, with the bottom two layers being kept fixed in their initial positions.

Supplementary note 2 Generalized Coordination Number (GCN)

The Generalized Coordination Number (GCN), a structure-sensitive descriptor proposed by Calle-Vallejo et al., characterizes the local structure-dependent trend of adsorption energies on surfaces as well as nanoparticles. Since the conventional coordination number only counts the number of nearest-neighboring atoms, it does not take into consideration the local environment of the active sites. By additionally incorporating the coordination of neighboring atoms, GCN is capable of discerning between seemingly similar, but genuinely different adsorption sites. GCN of an active site i with n_i first nearest neighbors is calculated as,

$$GCN(i) = \sum_{j=1}^{n_i} \frac{CN(j)}{CN_{max}} \qquad (1)$$

where $CN(j)$ is the coordination number of the nearest neighbor j and CN_{max} is the maximum coordination number of sites in bulk structures. For example, CN_{max} of a single top, 2-fold bridge, 3-fold hollow and 4-fold hollow sites in FCC crystal are 12, 18, 22 and 26, respectively.

Supplementary Figures

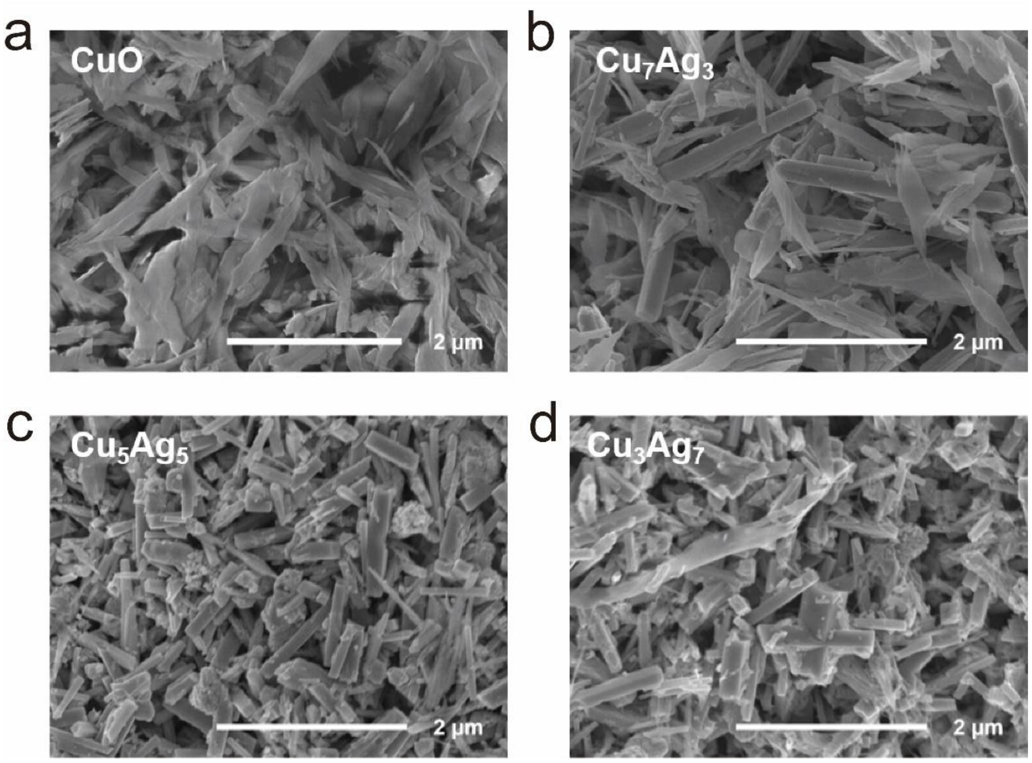

Supplementary Figure 1. Representative SEM images of as-prepared CuO and CuAg samples. (a) CuO samples, (b) Cu_7Ag_3 samples, (c) Cu_5Ag_5 samples, (d) Cu_3Ag_7 samples.

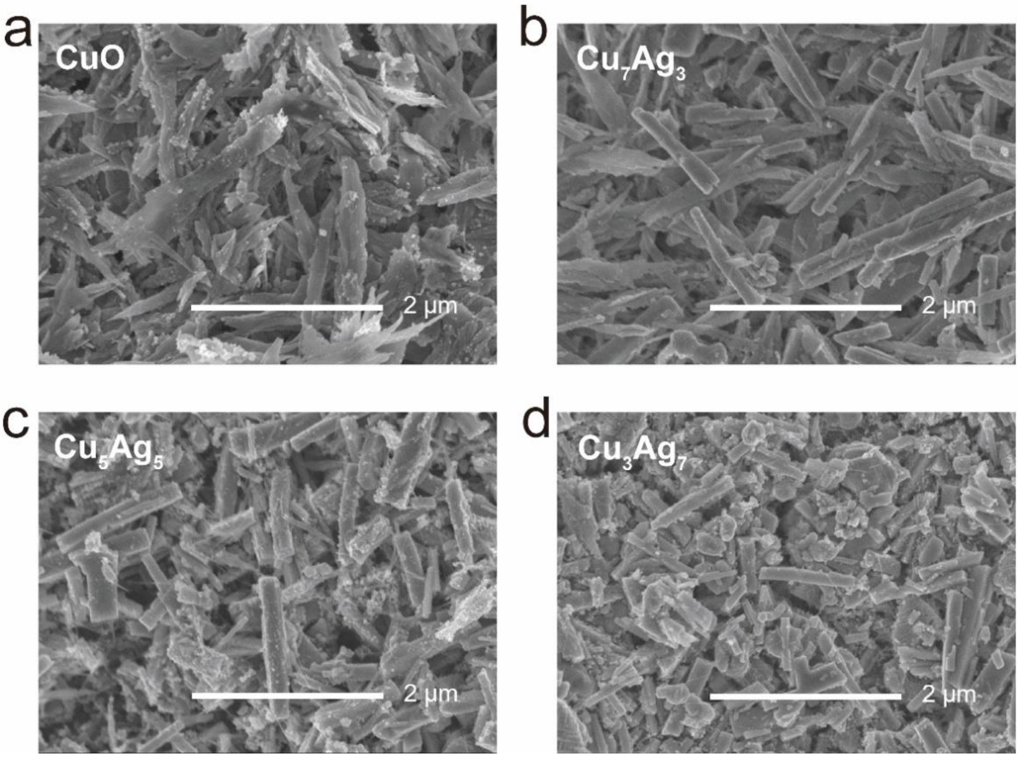

Supplementary Figure 2. Representative SEM images of post-reacted CuO and CuAg samples. (a) CuO samples, (b) Cu_7Ag_3 samples, (c) Cu_5Ag_5 samples, (d) Cu_3Ag_7 samples.

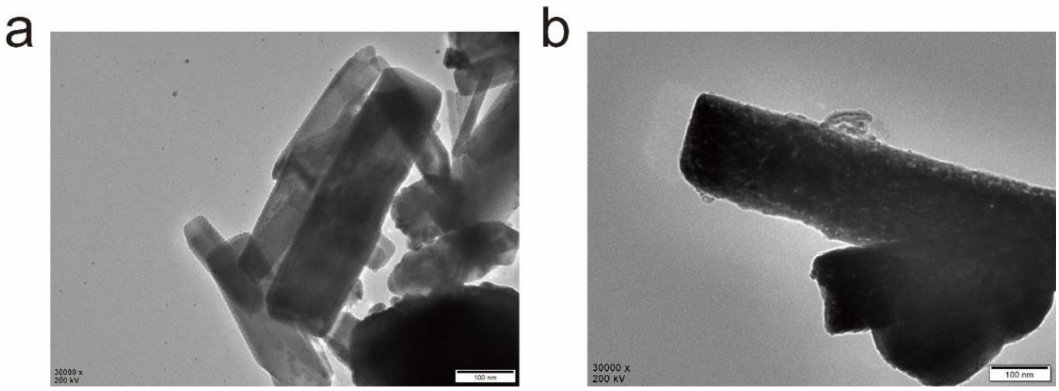

Supplementary Figure 3. TEM images of Cu_3Ag_7 samples. TEM images of (a) as-prepared Cu_3Ag_7 samples and (b) post-COR Cu_3Ag_7 samples.

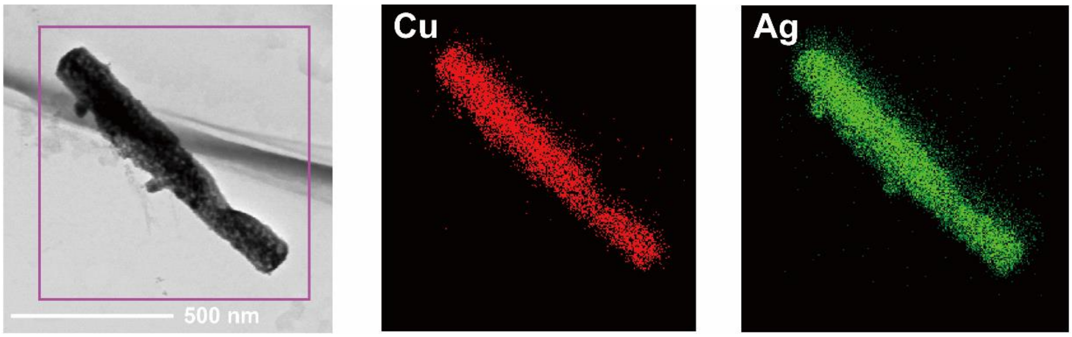

Supplementary Figure 4. TEM EDS-mapping of post-reacted Cu_5Ag_5 samples

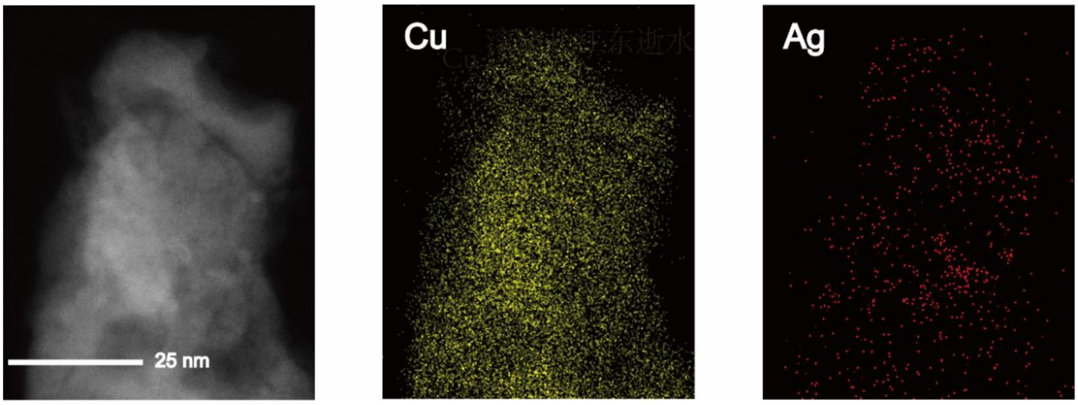

Supplementary Figure 5. STEM EDS-mapping of post-reacted Cu_7Ag_3 samples

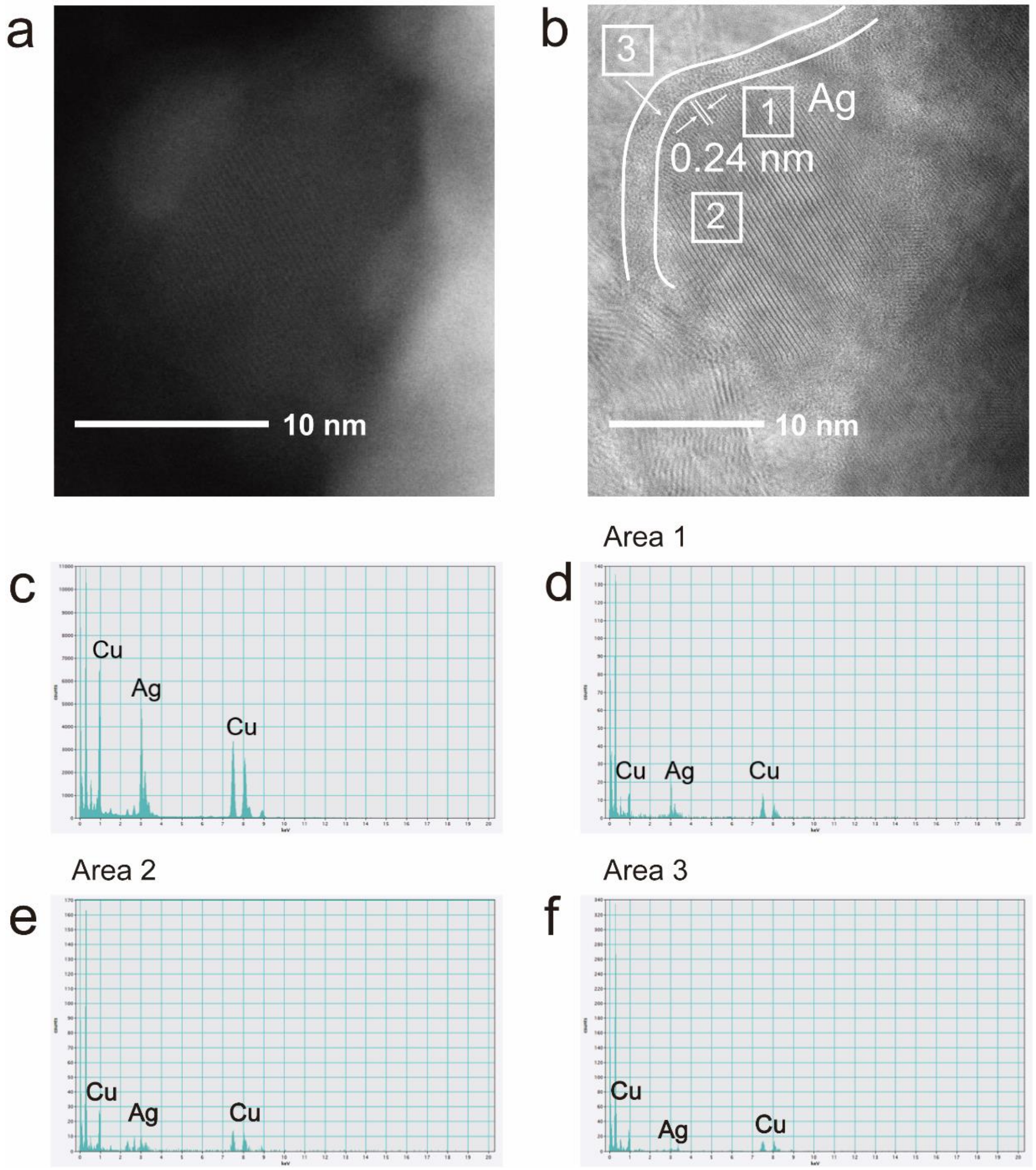

Supplementary Figure 6. Structural characterization of Cu_3Ag_7 catalysts after COR at 200 mA cm^{-2}. (a,b) HRTEM images of post-COR Cu_3Ag_7 samples. STEM EDS-mapping information of (c) the entire area, (d) area 1, (e) area 2 and (f) area 3.

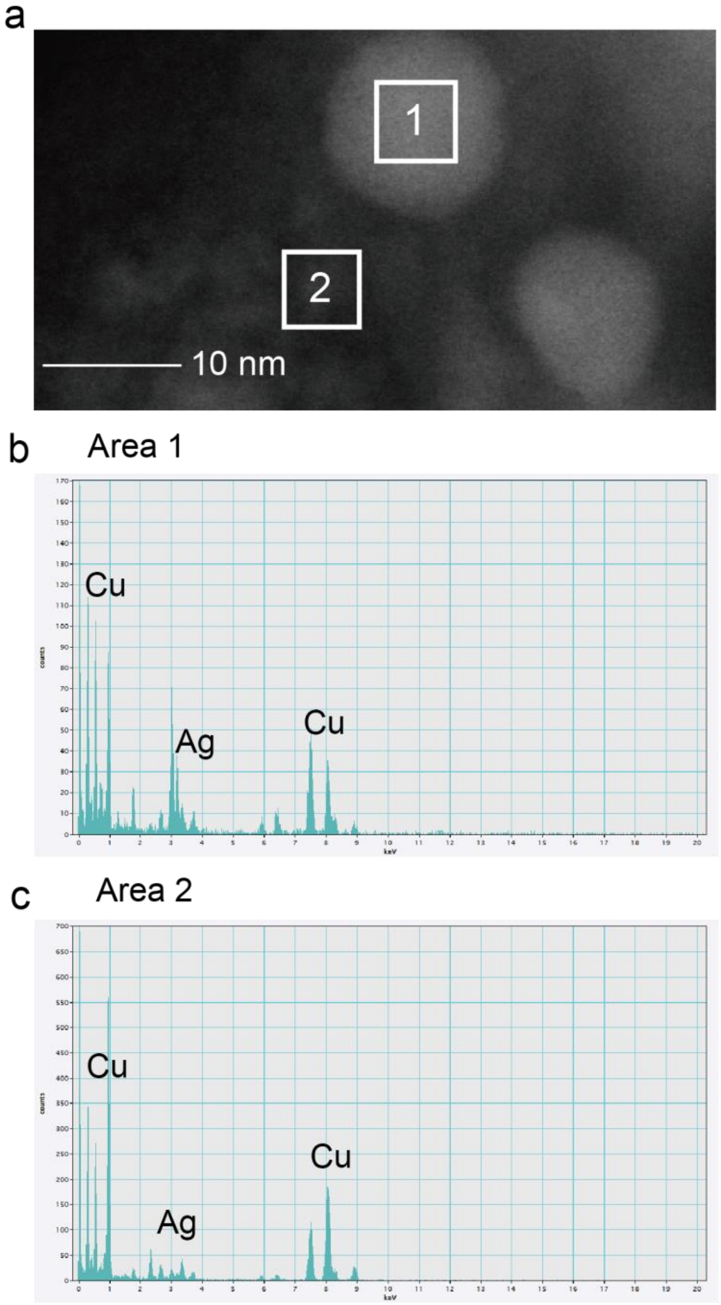

Supplementary Figure 7. Structural characterization of Cu_3Ag_7 catalysts after COR at 200 mA cm^{-2}. (a) HRTEM images of post-COR Cu_3Ag_7 samples. STEM EDS-mapping information of (b) area 1 and (c) area 2.

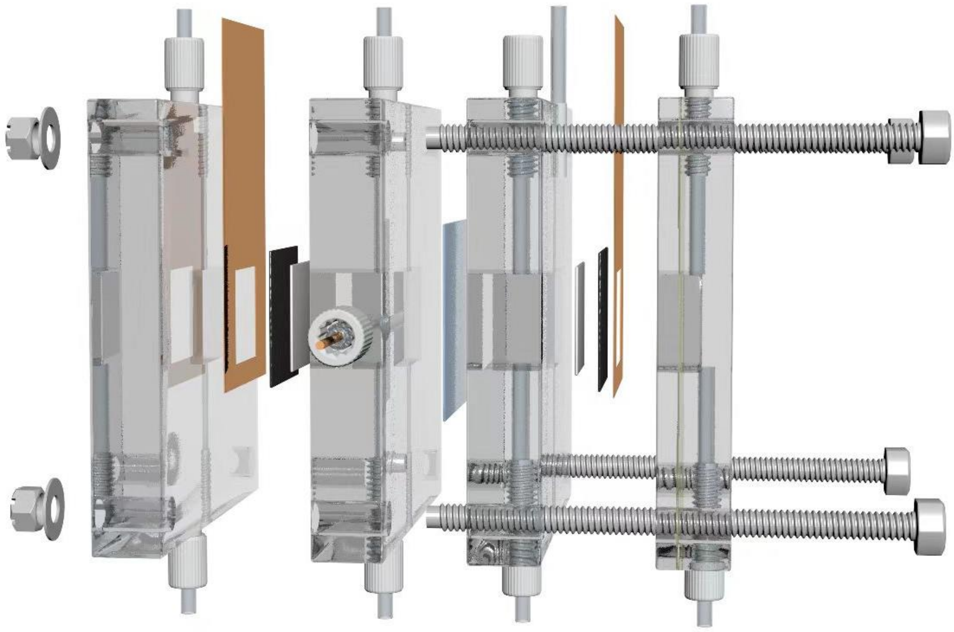

Supplementary Figure 8. Model figure of self-designed flow cell for COR/CO_2R

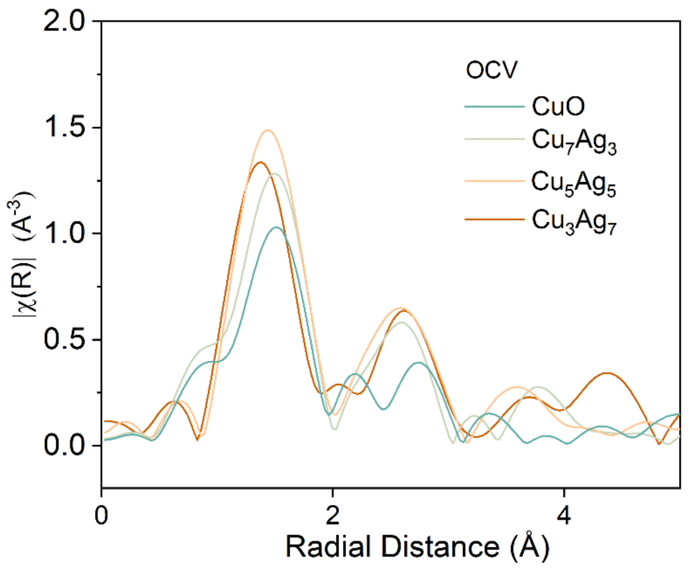

Supplementary Figure 9. FT of EXAFS spectra of CuO and CuAg samples at OCV

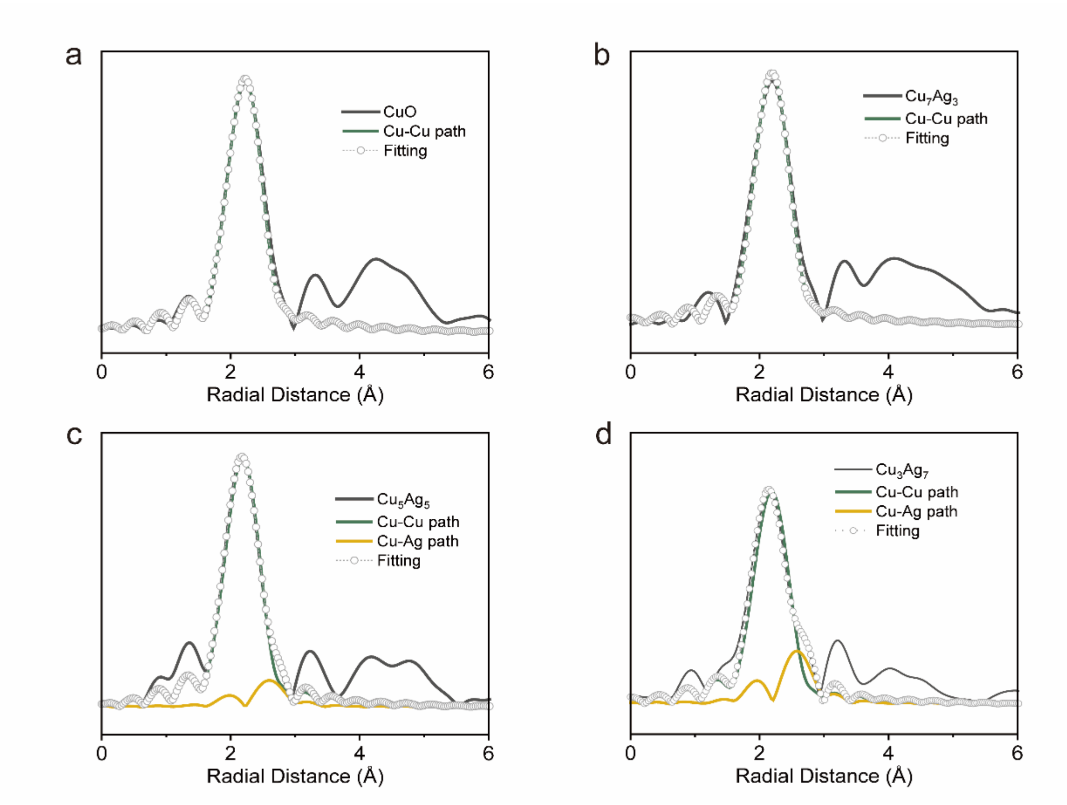

Supplementary Figure 10. *Operando* EXAFS fitting curves. (a) CuO, (b) Cu_7Ag_3, (c) Cu_5Ag_5, (d) Cu_3Ag_7 samples at −0.65 V vs RHE of COR.

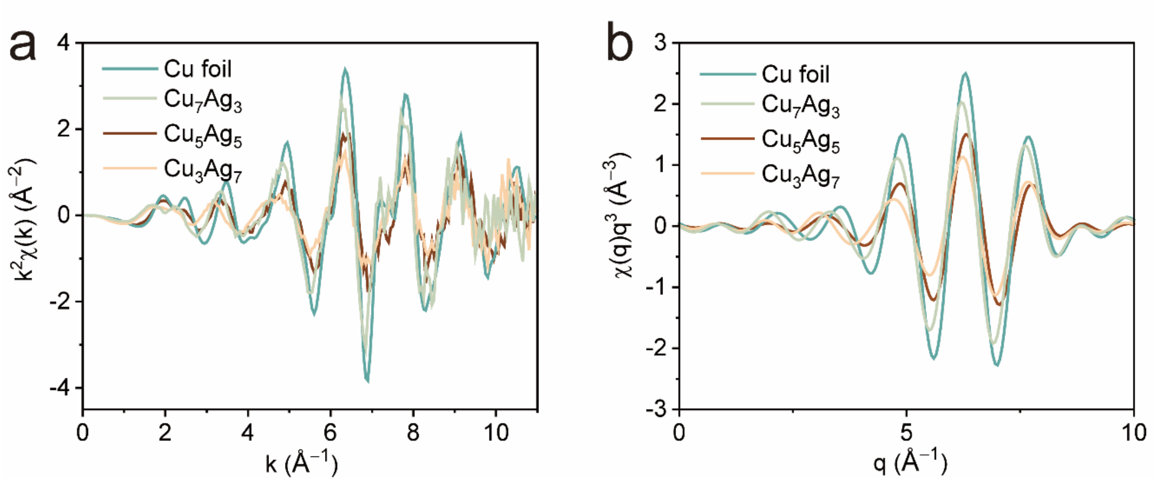

Supplementary Figure 11. EXAFS spectra of CuAg samples at −0.65 V vs RHE under COR and Cu foil in (a) k-space and (b) q-space.

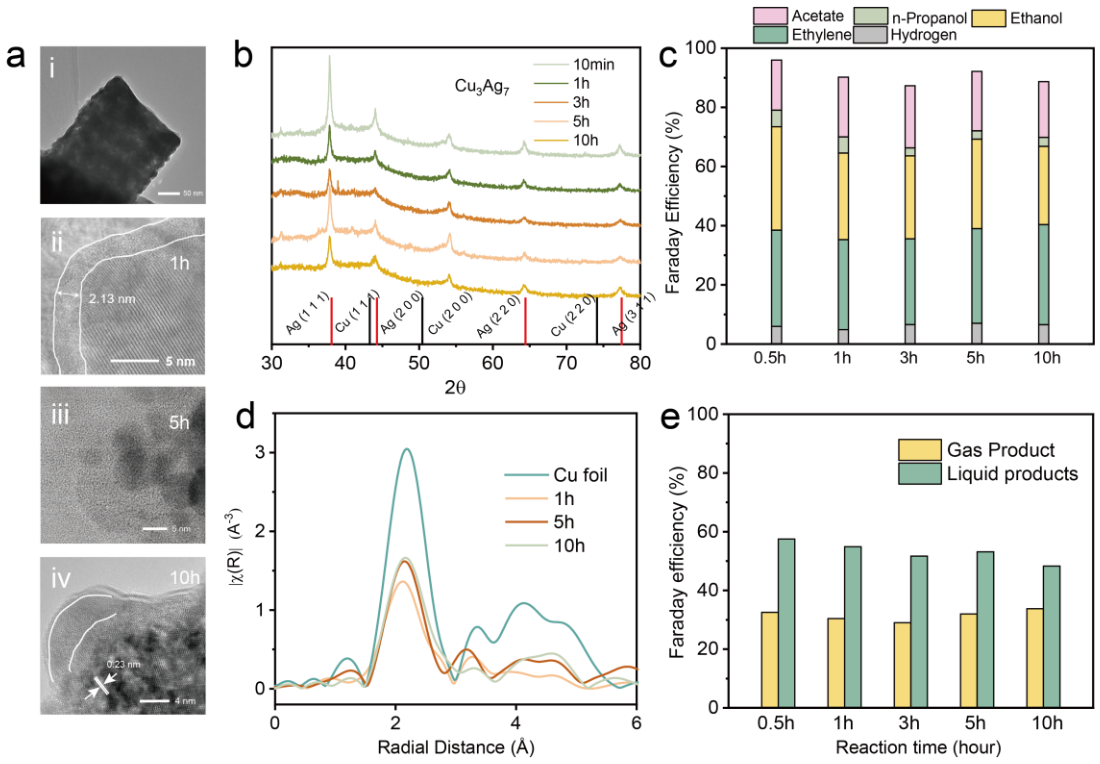

Supplementary Figure 12. COR performance and structural information of CuAg samples. (a) HRTEM images of rod-shaped particles edge in post-reacted Cu_3Ag_7. (b) XRD patterns of post-reacted Cu_3Ag_7 catalysts for 10min, 1, 3, 5 and 10h. (c) FEs of hydrogen, ethylene, acetate, ethanol and propanol during COR at 200 mA cm^{-2} on Cu_3Ag_7 catalysts for 0.5, 1, 3, 5 and 10 h. (d) FT of EXAFS spectra of Cu_3Ag_7 samples at −0.58 V vs RHE and standard Cu foil. (e) Total FEs of gas products and liquid products at 200 mA cm^{-2} on Cu_3Ag_7 catalysts for 0.5, 1, 3, 5 and 10 h.

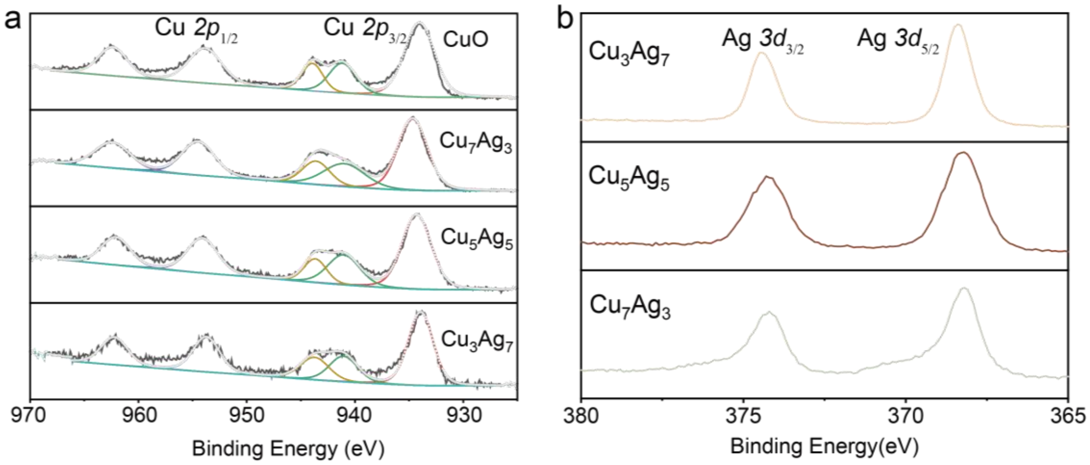

Supplementary Figure 13. XPS spectra of as-prepared samples. XPS spectra of CuO and CuAg samples on (a) Cu *2p* and (b) Ag *3d*.

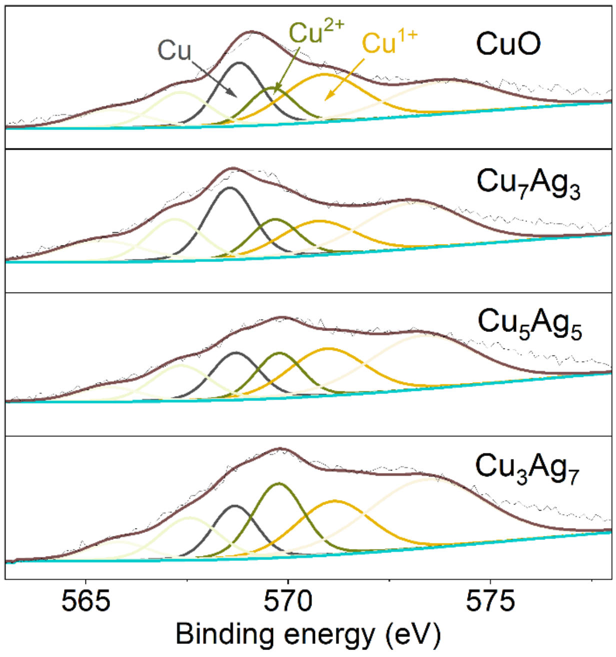

Supplementary Figure 14. XPS spectra of Cu LMM spectra of CuO and CuAg samples after COR with 200 mA cm^{-2} for 1h.

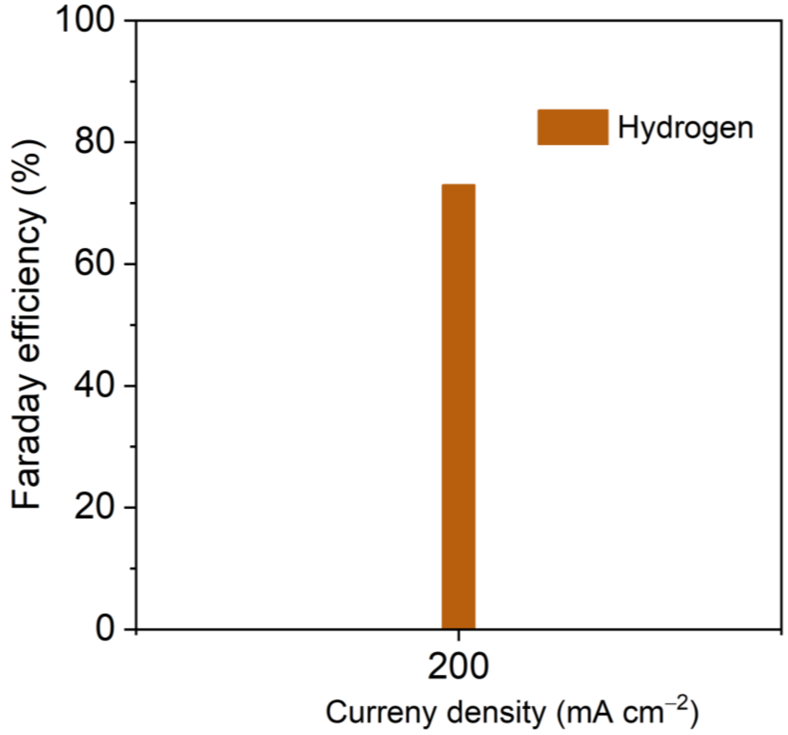

Supplementary Figure 15. Electrochemical performance of CO reduction on Ag_2O.

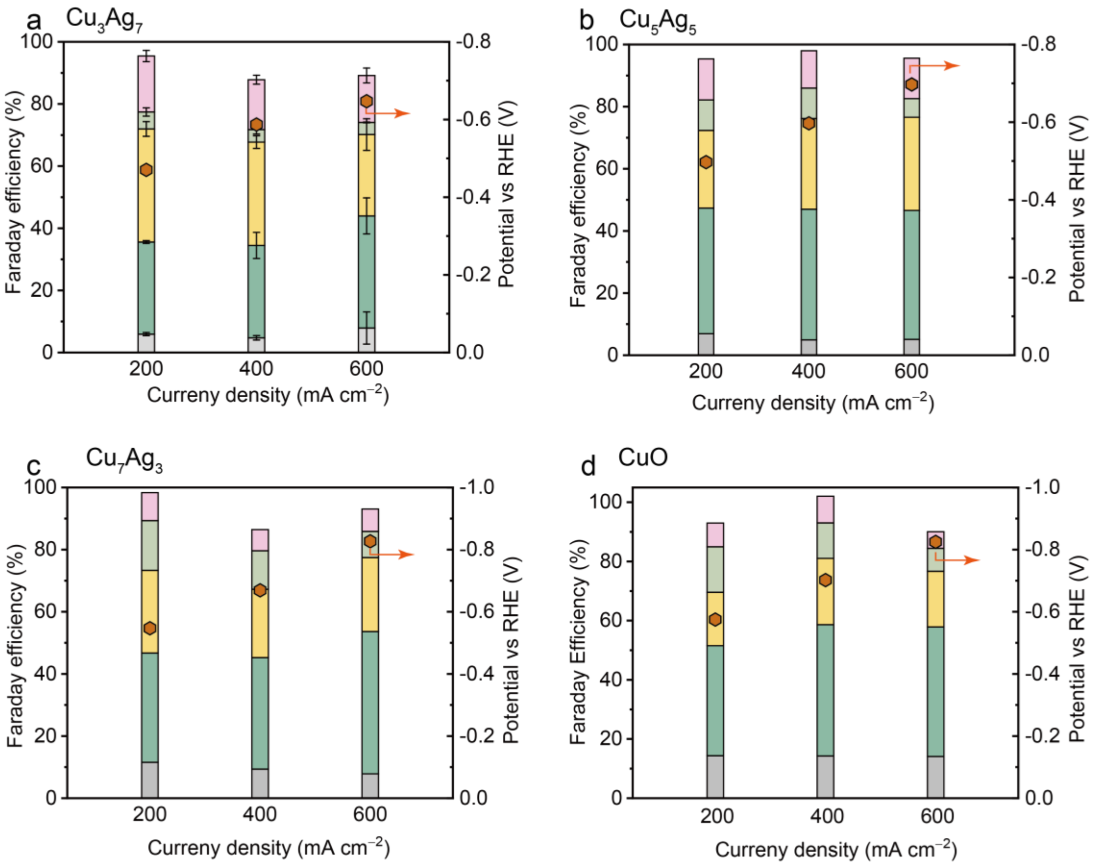

Supplementary Figure 16. Electrochemical performance of CO reduction. COR performance on (a) Cu_3Ag_7, (b) Cu_5Ag_5, (c) Cu_7Ag_3 and (d) CuO samples.

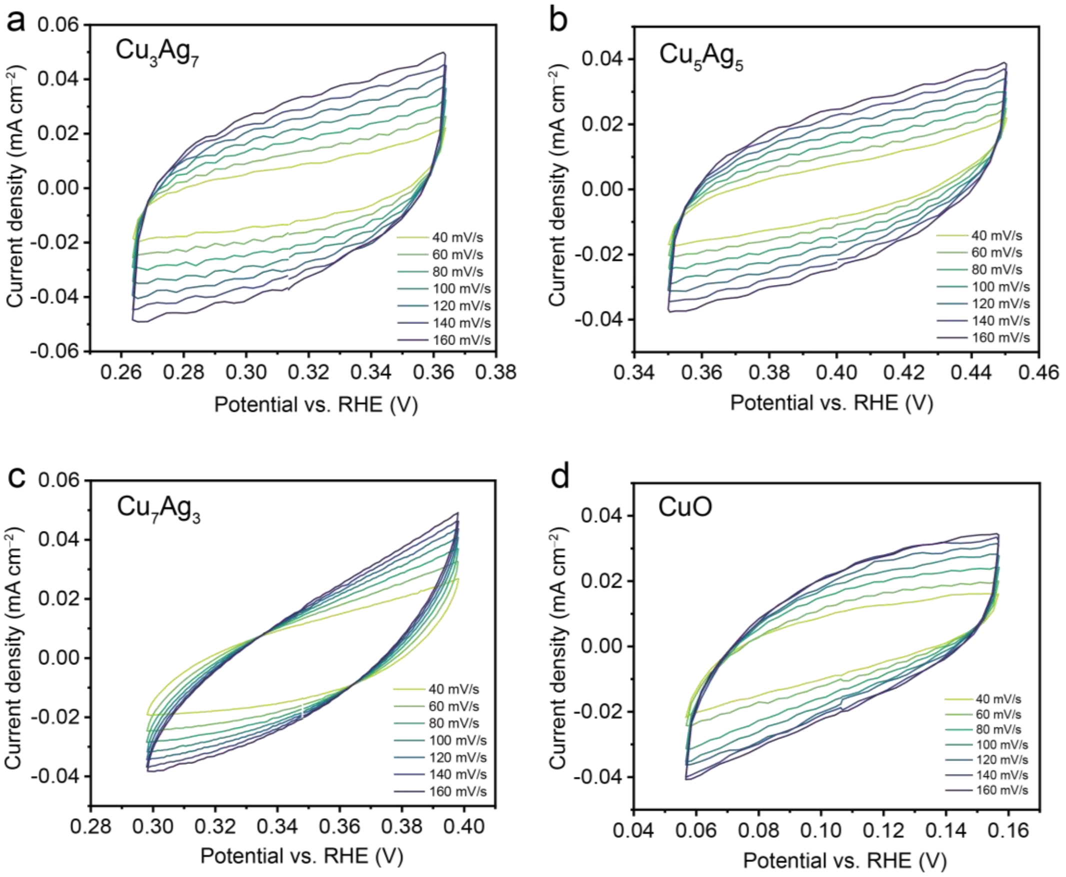

Supplementary Figure 17. Effective electrochemical active surface area tests (ECSA). ECSA of (a) Cu_3Ag_7, (b) Cu_5Ag_5, (c) Cu_7Ag_3 and (d) CuO samples.

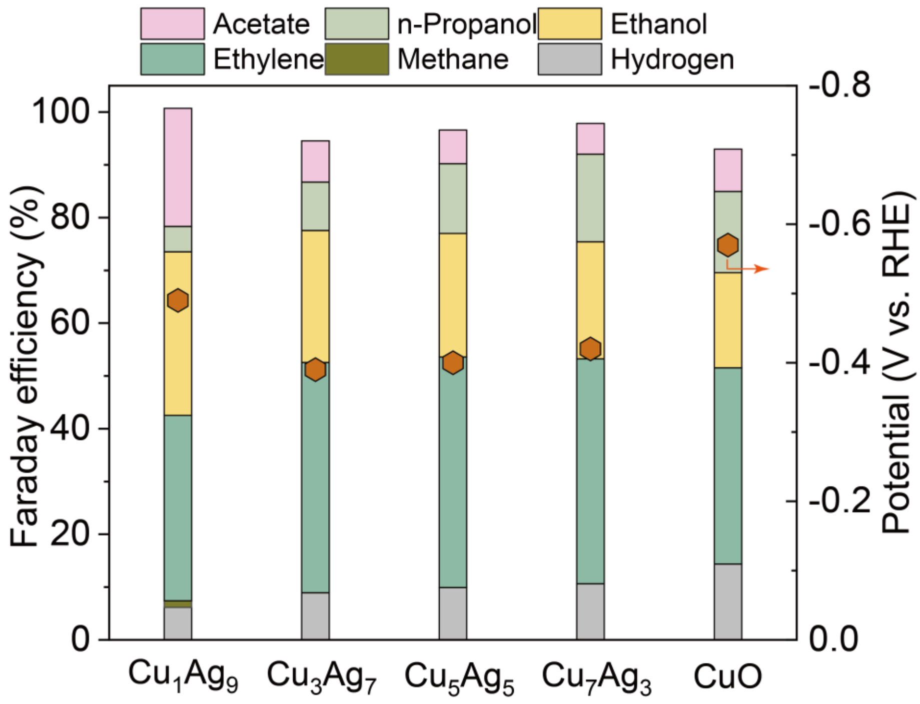

Supplementary Figure 18. Electrochemical performance of COR on CuO and physical-mixed CuAg samples at 200 mA cm^{-2}.

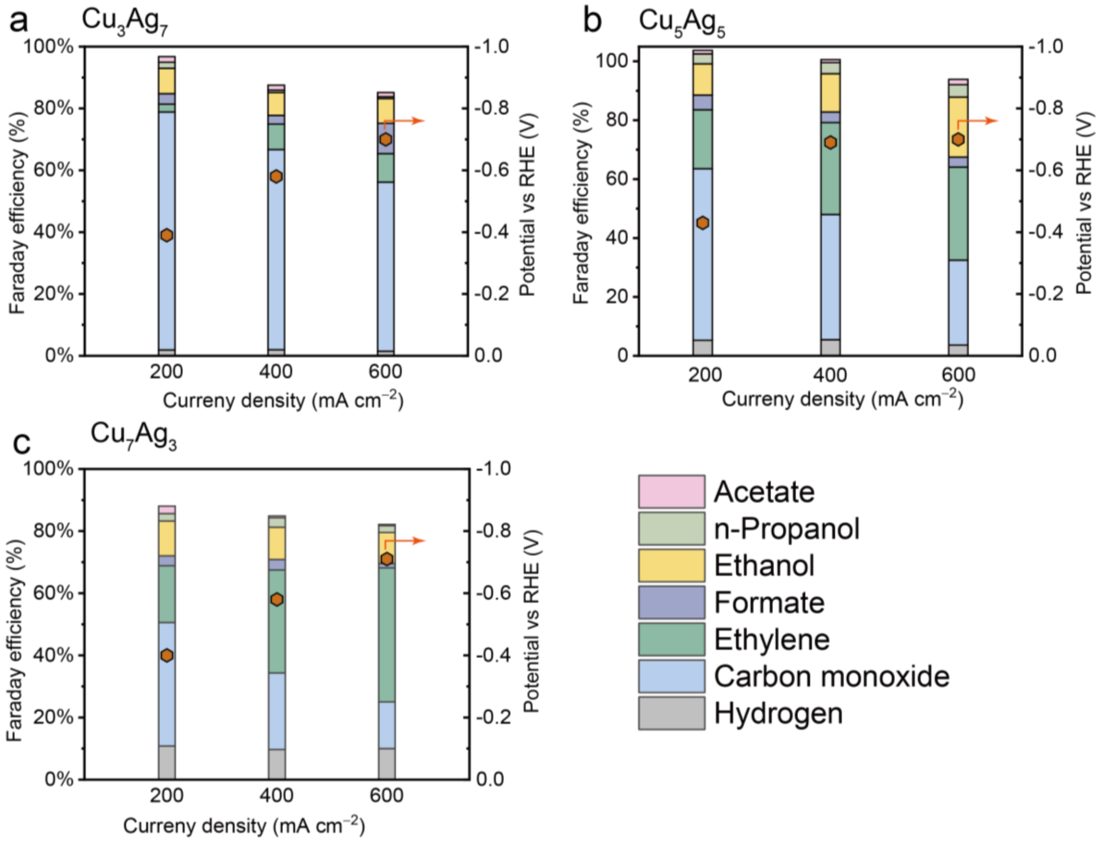

Supplementary Figure 19. Electrochemical performance of CO_2 reduction. CO_2R performance on (a) Cu_3Ag_7, (b) Cu_5Ag_5 and (c) Cu_7Ag_3 samples.

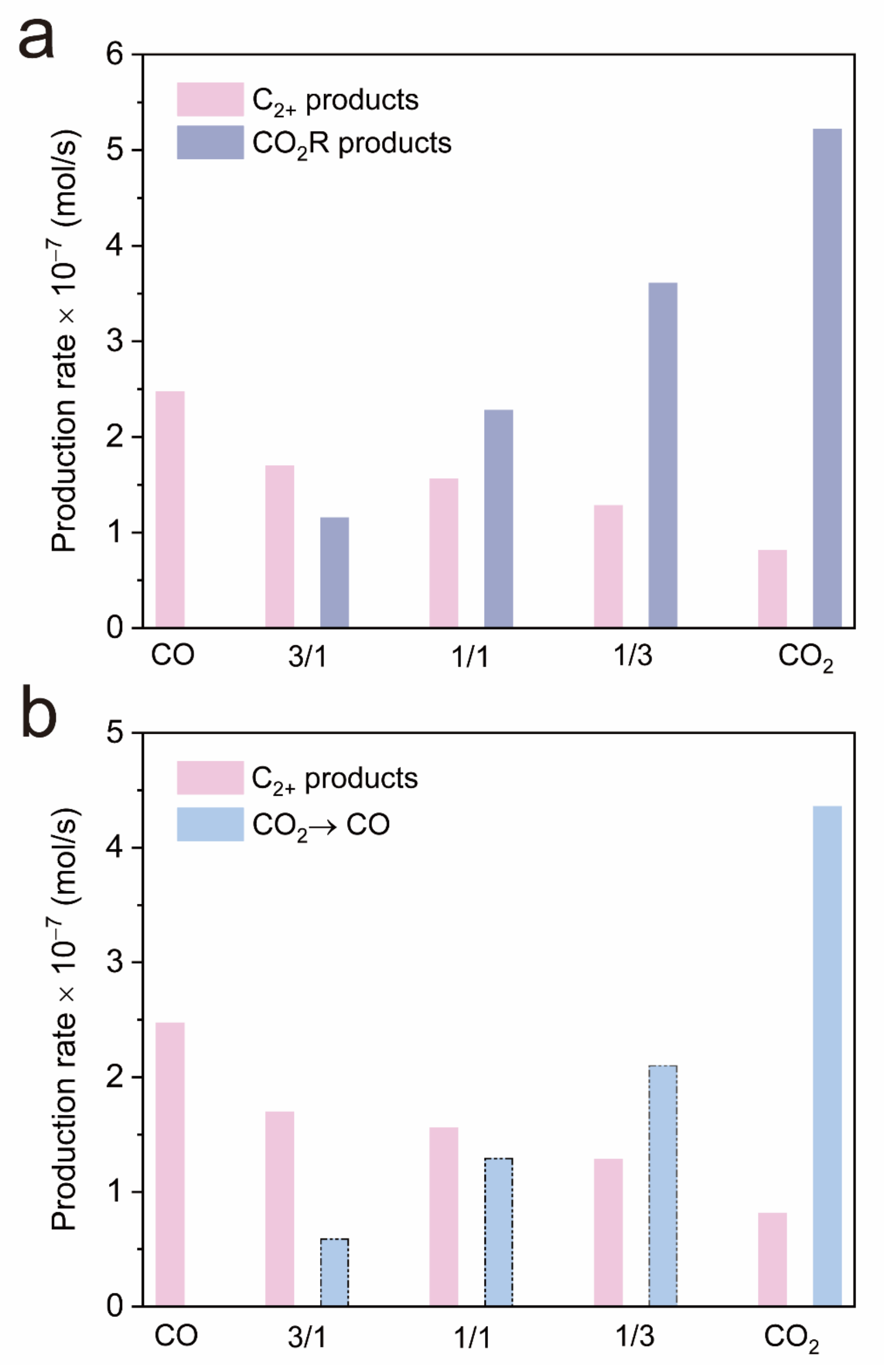

Supplementary Figure 20. Production rates across various feed-gas ratios under 200 mA cm^{-2}. Production rates on CuO of (a) C_{2+} products and CO_2R products and (b) C_{2+} products and CO from CO_2R.

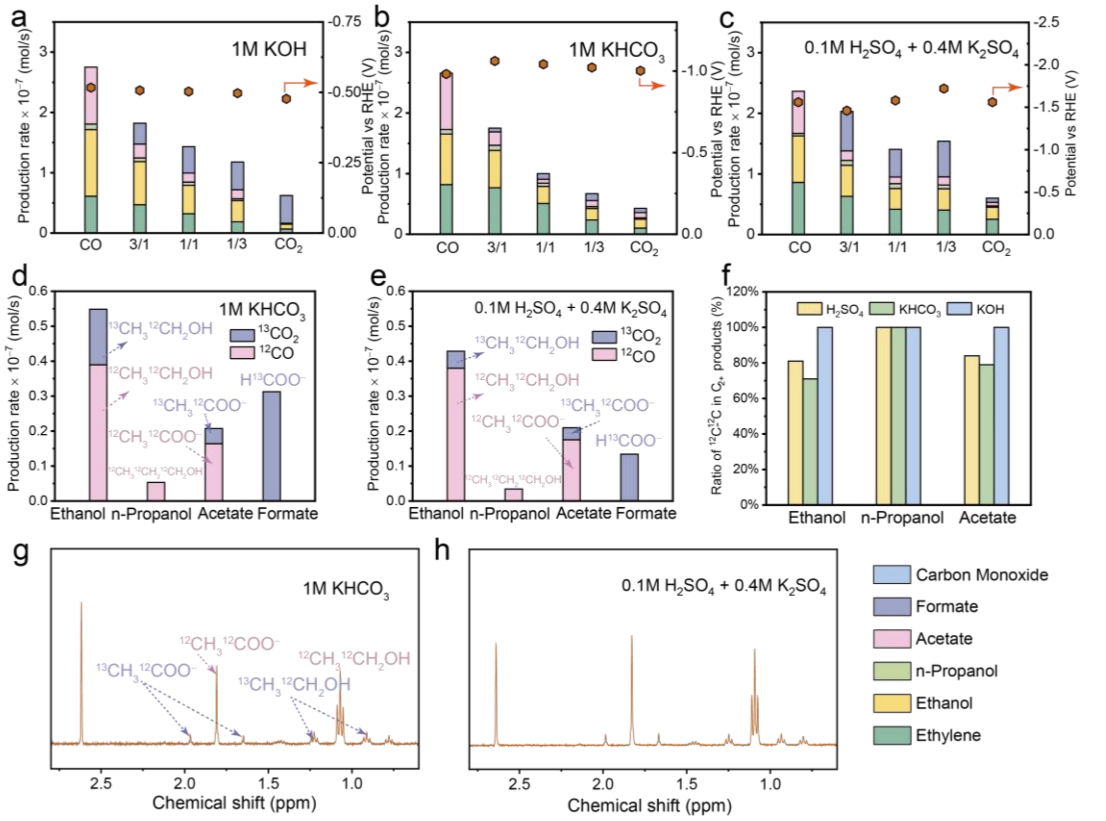

Supplementary Figure 21. CO/CO_2 co-feeding electrolysis and isotopic labelling experiments. Production rates of hydrogen, carbon monoxide, ethylene, acetate, ethanol and propanol on Cu_3Ag_7 across various feed-gas ratios under 200 mA cm^{-2} in (a) 1M KOH, (b) 1M $KHCO_3$ and (c) 0.1M H_2SO_4+0.4M K_2SO_4. Product distribution on Cu_3Ag_7 using isotopic labeled stream of $^{13}CO_2$/^{12}CO with 1/1 ratio under 200 mA cm^{-2} in (d) 1M $KHCO_3$ and (e) 0.1M H_2SO_4+0.4M K_2SO_4. (f) Ratio of ^{12}CO in C_{2+} liquid products in different electrolytes under 200 mA cm^{-2}. ^{1}H-NMR spectra on Cu_3Ag_7 samples in (g) 1M $KHCO_3$ and (h) 0.1M H_2SO_4+0.4M K_2SO_4 at 200 mA cm^{-2}.

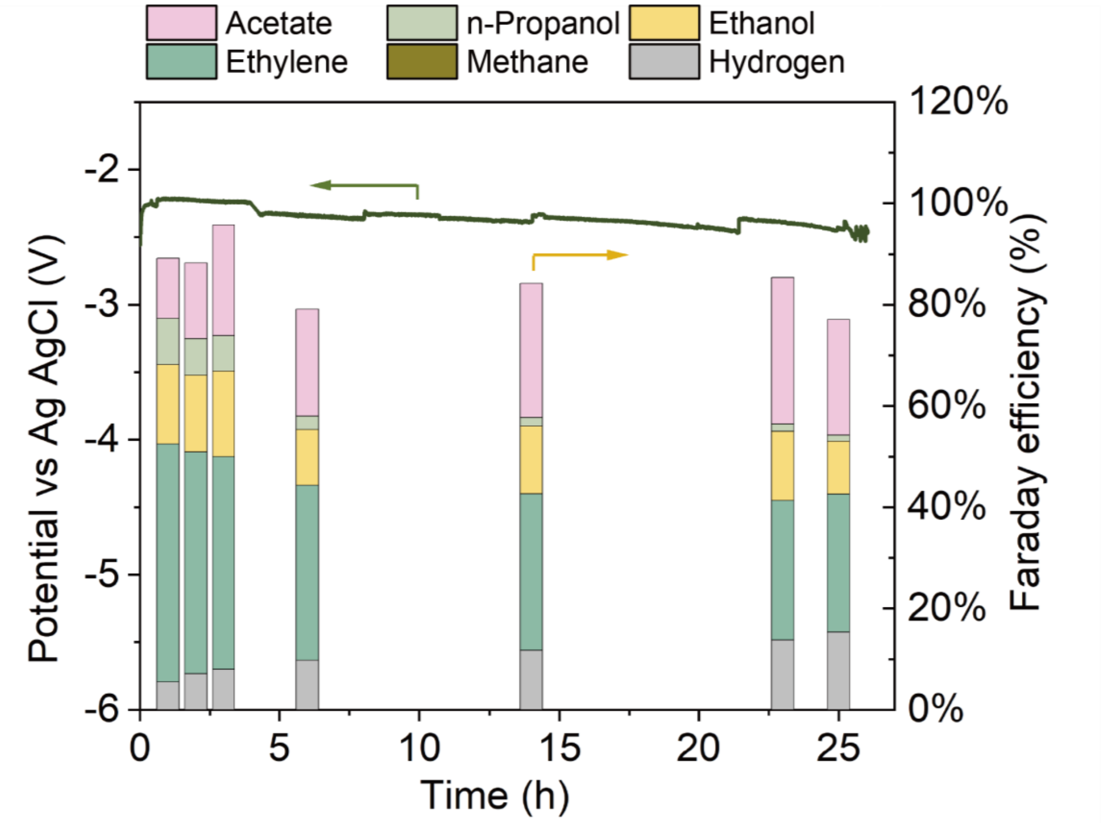

Supplementary Figure 22. Potential and FEs of ethylene and hydrogen on Cu_5Ag_5 samples in long-time test

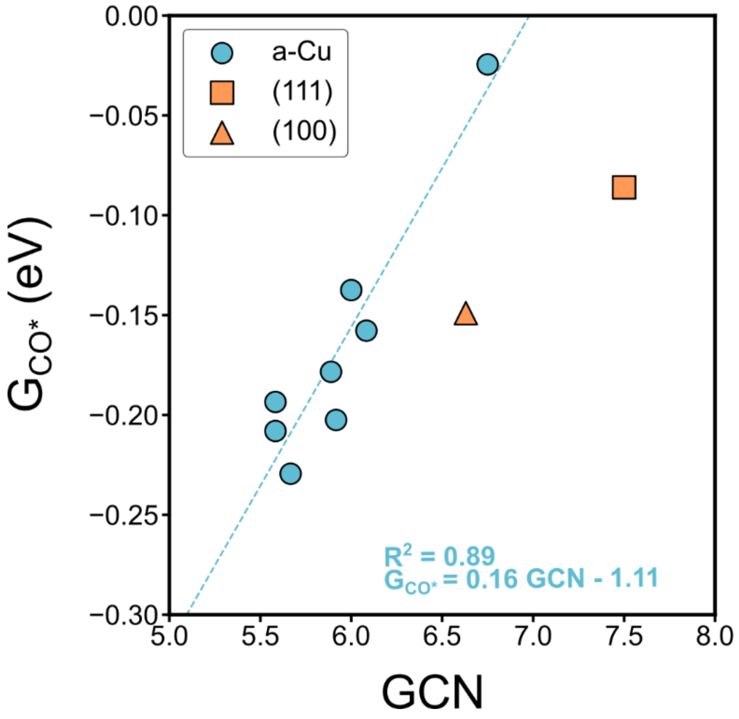

Supplementary Figure 23. Binding free energies on amorphous Cu and Cu(111) and (100). The binding free energies of CO* (G_{CO^*}) on a-Cu surface plotted with respect to GCN. Stronger binding of CO* on a-Cu was observed compared to the crystalline Cu (111) and (100) facets.

Supplementary Tables

Supplementary Table 1. ICP-OES analysis of Cu and Ag ratio for CuAg samples.

	Cu_7Ag_3	Cu_5Ag_5	Cu_3Ag_7
Cu/Ag ratio in as-prepared samples	2.36	1.02	0.43
Cu/Ag ratio in post-reacted samples	2.27	1	0.39

Supplementary Table 2. ICP-OES analysis of Cu and Ag in electrolyte for CuAg samples.

Samples		Concentration(ppm)	Weight (mg)
Cu_7Ag_3	Cu	0.08	8×10^{-4}
	Ag	0.1352	1.35×10^{-3}
Cu_5Ag_5	Cu	0.2626	2.62×10^{-3}
	Ag	0.2166	2.17×10^{-3}
Cu_3Ag_7	Cu	0.5126	5.13×10^{-3}
	Ag	0.2106	2.11×10^{-3}

Supplementary Table 3. Fitting parameters of Fourier transform (FT) of EXAFS on CuO samples.

	Path	Coordination number	$S0^2$	R (Å)	R-factor
**CuO**	Cu-Cu	8.14 ± 0.88	0.92	2.54	0.009

Supplementary Table 4. Fitting parameters of Fourier transform (FT) of EXAFS on Cu_7Ag_3 samples.

	Path	Coordination number	$S0^2$	R (Å)	R-factor
**Cu_7Ag_3**	Cu-Cu	9.95 ± 1.47	0.92	2.54	0.011

Supplementary Table 5. Fitting parameters of Fourier transform (FT) of EXAFS on Cu_5Ag_5 samples.

	Path	Coordination number	$S0^2$	R (Å)	R-factor
**Cu_5Ag_5**	Cu-Cu	6.76 ± 1.74	0.92	2.52	0.028
	Cu-Ag	0.75 ± 0.84		2.68	

Supplementary Table 6. Fitting parameters of Fourier transform (FT) of EXAFS on Cu_3Ag_7 samples.

	Path	Coordination number	$S0^2$	R (Å)	R-factor
**Cu_3Ag_7**	Cu-Cu	6.11 ± 1.33	0.92	2.53	0.024
	Cu-Ag	1.52 ± 0.69		2.68	

Supplementary Table 7. GCN of various adsorption sites on Cu facets.

Facet	Adsorption Site	GCN
(111)	single top	7.5
(111)	2-fold bridge	7.33
(111)	3-fold hollow	6.95
(100)	single top	6.67
(100)	2-fold bridge	6.67
(100)	4-fold hollow	6.61
